# Supplementary material for: Cell cycle dynamics regulate H3K27 and H3K9 histone modifications in Drosophila
Source: PLoS Biol. 2026 Mar 26;24(3):e3003371. doi: 10.1371/journal.pbio.3003371 (PMC13046271; doi:10.1371/journal.pbio.3003371)
Supplement: S1 Table — List of Drosophila strains, transgenes, antibodies, and software used in this study. Sources, providers and unique IDs are provided were available. (DOCX) [file pbio.3003371.s001.docx]

**Table S1 Experimental strains, reagents and software**

| **Tool** | **Source** | **ID** |
| --- | --- | --- |
|  |  |  |
| *w^118^* | David Bilder, University of California, Berkeley | FBal0018157 |
| *UAS-GFP^S56T^* | Bloomington Drosophila Stock Center | BDSC: 1521 |
| *rn^GAL4-DeltaS^, tubGAL80^ts^* | Bloomington Drosophila Stock Center | BDSC: 8142; 7018 recombinant |
| *rn^GAL4-5^, UAS-egr, tubP-GAL80^ts^* | Iswar Hariharan, University of California, Berkeley | BDSC: 8142; 7018 recombinant |
| *‘E2F1-FUCCI, CycB-FUCCI’; Ubi-GFP.E2f1^1-230^, Ubi-mRFP1.NLS.CycB^1-266^* | Bloomington Drosophila Stock Center | BDSC: 55123 |
| *UAS-Cdk1 RNAi [TRiP.HMS01531]* | Bloomington Drosophila Stock Center | BDSC: 36117 |
| *If/CyO-GFP; UAS-E2F, UAS-DP/TM6b* | Laura Buttitta, University of Michigan | PMID: 9657151 |
| *UAS-pnt.P1* | Bloomington Drosophila Stock Center | BDSC: 869 |
| *UAS-E(z)-RNAi (TRiP.HMS00066)* | Bloomington Drosophila Stock Center | BDSC: 33659 |
| *UAS-nejire-RNAi(TRiP.HMS01507)* | Bloomington Drosophila Stock Center | BDSC: 37489 |
| *UAS-InR-DA (UAS-InR.A1325D)* | Bloomington Drosophila Stock Center | BDSC: 8263 |
| *‘TRE-RFP’: TRE-DsRed.T4* | Dirk Bohmann, University of Rochester Medical Center | PMID: 22509270 |
| *UAS-nejire* | Bloomington Drosophila Stock Center | BDSC: 32573 |
| *UAS-HDAC1-RNAi (TRiP.HMS00607)* | Bloomington Drosophila Stock Center | BDSC: 33725 |
| *CBP-GFP* | Melissa Harrison, University of Wisconsin School of Medicine and Public Health | PMID: 40441155 |
| *UAS-HDAC1* | Bloomington Drosophila Stock Center | BDSC: 32242 |
| *UAS-dicer; UAS-CyclinE-RNAi/CyO, act>GFP* | Carlos Estella, Centro de Biología Molecular Severo Ochoa (CSIC-UAM), Universidad Autónoma de Madrid | VDRC ID: 110204 |
| *UAS-dacapo* | Carlos Estella, Centro de Biología Molecular Severo Ochoa (CSIC-UAM), Universidad Autónoma de Madrid |  |
|  |  |  |
| Chicken anti-GFP | Abcam | Cat. #: ab13970 |
| Rabbit monoclonal anti-GFP | Invitrogen | Cat. #: G10362 |
| Rat monoclonal anti-RFP | ChromoTek | Cat. #: 5F8 RRID: AB_2336064 |
| Rabbit polyclonal anti-H3K9ac | Active motif | Cat. #: 39017 RRID: AB_2616593 |
| Rabbit polyclonal anti-H4K8ac | Active motif | Cat. #: 61104  RRID: AB_2793506 |
| Rabbit polyclonal anti-acetyl Lysine | Abcam | Cat. #: ab80178 |
| Rabbit monoclonal anti-crotonyl Histone H3 | PTMab | Cat. #: PTM-517RM |
| Rabbit polyclonal anti-H3K9me1me2me3 | Active motif | Cat. #: 39242  RRID: AB_2793200 |
| Rabbit polyclonal anti-H3K18ac | Active motif | Cat. #: 39756  RRID: AB_2714186 |
| Mouse monoclonal anti-H3K27ac | Active motif | Cat. #: 39085  RRID: AB_2793305 |
| Rabbit polyclonal anti-H3K4me3 | Abcam | Cat. #: ab8580 |
| Mouse monoclonal anti-H3K27me3 | Abcam | Cat. #: ab6002 |
| Rabbit polyclonal anti-H3K9me3 | Abcam | Cat. #: ab8898 |
| Rabbit polyclonal anti-H3 | Abcam | Cat. #: ab1791 |
| Rabbit polyclonal anti-Histone H2AvD phosphoS137 | Rockland | Cat. #: 600-401-914 |
| Mouse monoclonal anti-Cut | DSHB | Cat. #: 2B10  RRID: AB_528186 |
| Mouse monoclonal anti-  Engrailed/Invected | DSHB | Cat. #: 4D9  RRID: AB_528224 |
| Mouse monoclonal anti-Nubbin | DSHB | Cat. #: 2D4  RRID: AB_2722119 |
| Mouse monoclonal anti-Antp | DSHB | Cat. #: 8C11  RRID: AB_528083 |
| Mouse monoclonal anti-Ptc | DSHB | Cat. #: Apa1  RRID: AB_528441 |
| Mouse monoclonal anti-Wg | DSHB | Cat. #: 4D4  RRID: AB_528512 |
| Mouse monoclonal anti-Histone H3 (for CUT&Tag) | Active motif | Cat. #: 39763  RRID: AB_2650522 |
| Rabbit polyclonal anti-H3K27ac (for CUT&Tag) | Diagenode | Cat. #: C15410196 |
| Rabbit polyclonal anti-H3K27me3 (for CUT&Tag) | Diagendoe | Cat. #: C15410195 |
| Rabbit monoclonal anto-H3K9me3 (for CUT&Tag) | Abcam | Cat. #: ab176916 |
| DAPI | Sigma Aldrich | Cat #: D9564 |
| Goat anti-mouse IgG Alexa Flour 488 | Invitrogen | Cat. #: A-11001 |
| Goat anti-chicken IgY Alexa Flour 488 | Invitrogen | Cat. #: A-11039 |
| Goat anti-rabbit IgG Alexa Flour 488 | Invitrogen | Cat. #: A-11008 |
| Goat anti-mouse IgG Alexa Flour 555 | Invitrogen | Cat. #: A-21422 |
| Goat anti-rat IgG Alexa Flour 555 | Invitrogen | Cat. #: A-21434 |
| Goat anti-mouse Alexa Flour 647 | Invitrogen | Cat. #: A-21235 |
| Donkey anti-mouse Alexa Flour 647 preabsorbed | Abcam | Cat. #: ab150111 |
| Goat anti-rat Alexa Flour 647 | Invitrogen | Cat. #: A-21247 |
| Goat anti-rabbit Alexa Flour 647 | Invitrogen | Cat. #: A-21244 |
| Click-iT Plus EdU Alexa Fluor 647 Imaging Kit | Invitrogen | Cat. #: C10640 |
| Click-iT Plus OPP Alexa Fluor 647 Imaging Kit | Invitrogen | Cat. #: C10458 |
| CellEvent Senescence Green Detection Kit | Invitrogen | Cat. #: C10850 |
|  |  |  |
| FIJI (ImageJ 1.54f) | (Schindelin et al., 2012) | https://fiji.sc/ |
| GraphPad Prism | Graph Pad | version 9.5.0 |
| Affinity Designer | Affinity | Up to version 2.6.3 |
| Biorender | Science Suite Inc. DBA BioRender | Biorender |
