# Supplementary figures and images for: Cell cycle dynamics regulate H3K27 and H3K9 histone modifications in Drosophila
Source: PLoS Biol. 2026 Mar 26;24(3):e3003371. doi: 10.1371/journal.pbio.3003371 (PMC13046271; doi:10.1371/journal.pbio.3003371)

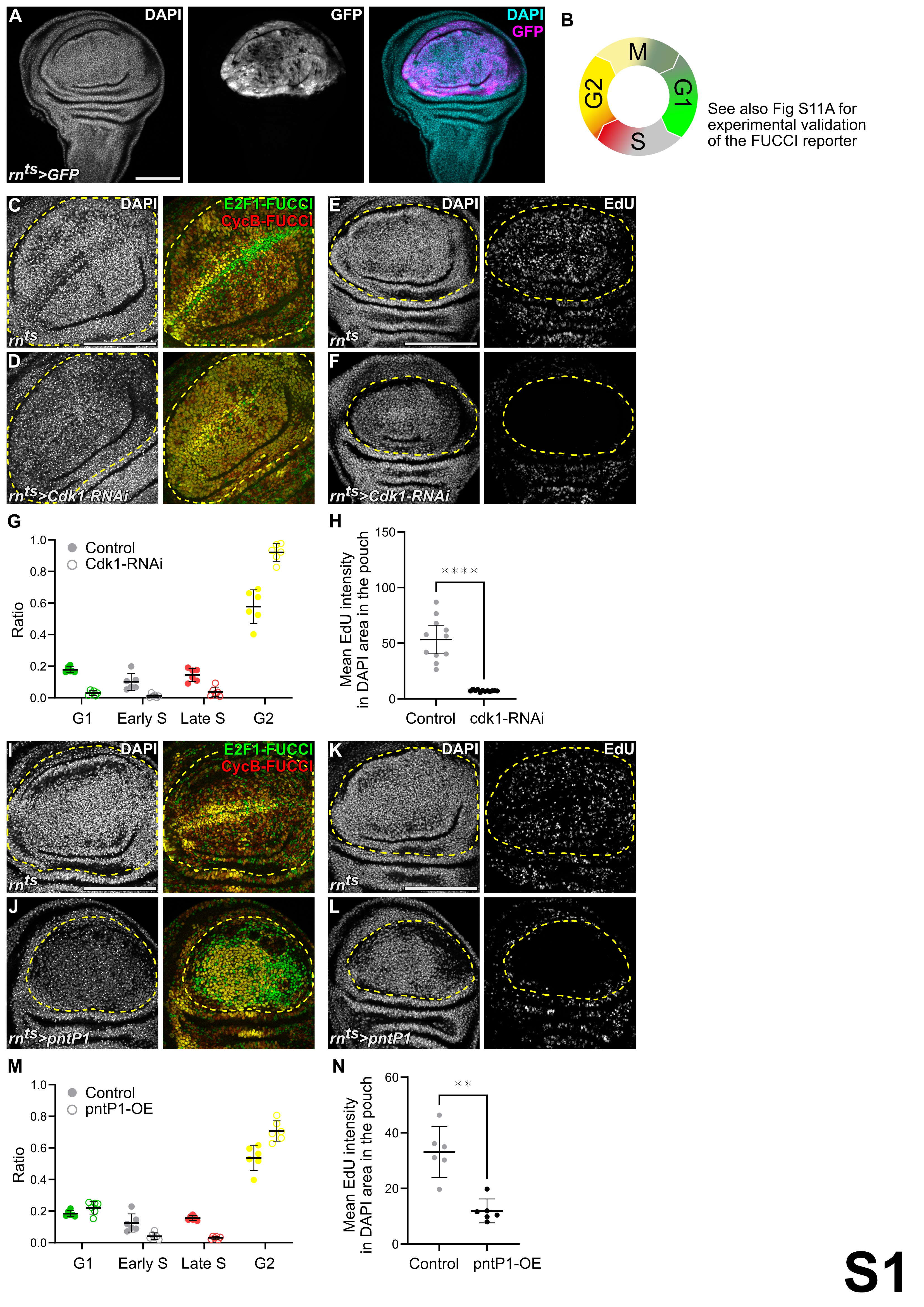

Supplement: S1 Fig — A. A control wing disc after 24 h of UAS-GFP-expression in the pouch, under the control of the rn-GAL4 (rotund-GAL4) driver. GFP-expression visualizes the tissue domain subject to manipulation throughout this study (magenta). B. Schematic representation of the Fly-FUCCI cell cycle reporter system, utilizing the degradable GFP-E2F11-230 (green) and mRFP-NLS-CycB1-266 (red) as sensors to visualize cell cycle phases. Individual fluorophore expression indicates cells in G1 or late S-phase, respectively. Combined expression of both GFP and RFP labels cells is observed in early and late G2. Cells in late mitosis and early S-phase lack expression of either GFP or RFP. Cells in early S-phase are specifically detected by EdU incorporation [63,66]. Cells in mitosis are positioned in very apical positions of the tissue and are not represented in our study. C–F. Cell cycle dynamics in control discs (C, E) and discs expressing Cdk1-RNAi for 24 h under the control of rn-GAL4 (D, F). FUCCI reporters GFP-E2F11-230 (green) and mRFP-NLS-CycB1-266 (red) were used to visualize cell cycle phases (C, D) and EdU incorporation was used to detect DNA replication in S-phase cells (E, F). Cdk1 knockdown in the wing pouch domain leads to a cell cycle phase shift toward G2 phase (D), and a corresponding loss of EdU incorporation (F) combined demonstrating a pronounced arrest in G2. G. Quantified cell cycle phase ratios in control and Cdk1-RNAi-expressing discs (see Materials and Methods for details). Each dot represents one disc; symbols/colors denote the indicated genotypes and cell cycle phases. Error bars indicate mean ± SD. H. Quantification of mean EdU intensity per DAPI area in the pouch region of control and Cdk1-RNAi-expressing discs, serving as a proxy for relative DNA replication activity. Mean and 95% CI are shown. Statistical significance was tested using two-tailed Welch’s test (control discs: n = 11, Cdk1-RNAi-expressing discs: n = 12). I–L. Cell cycle dynamics in control discs (I [file pbio.3003371.s003.tiff]

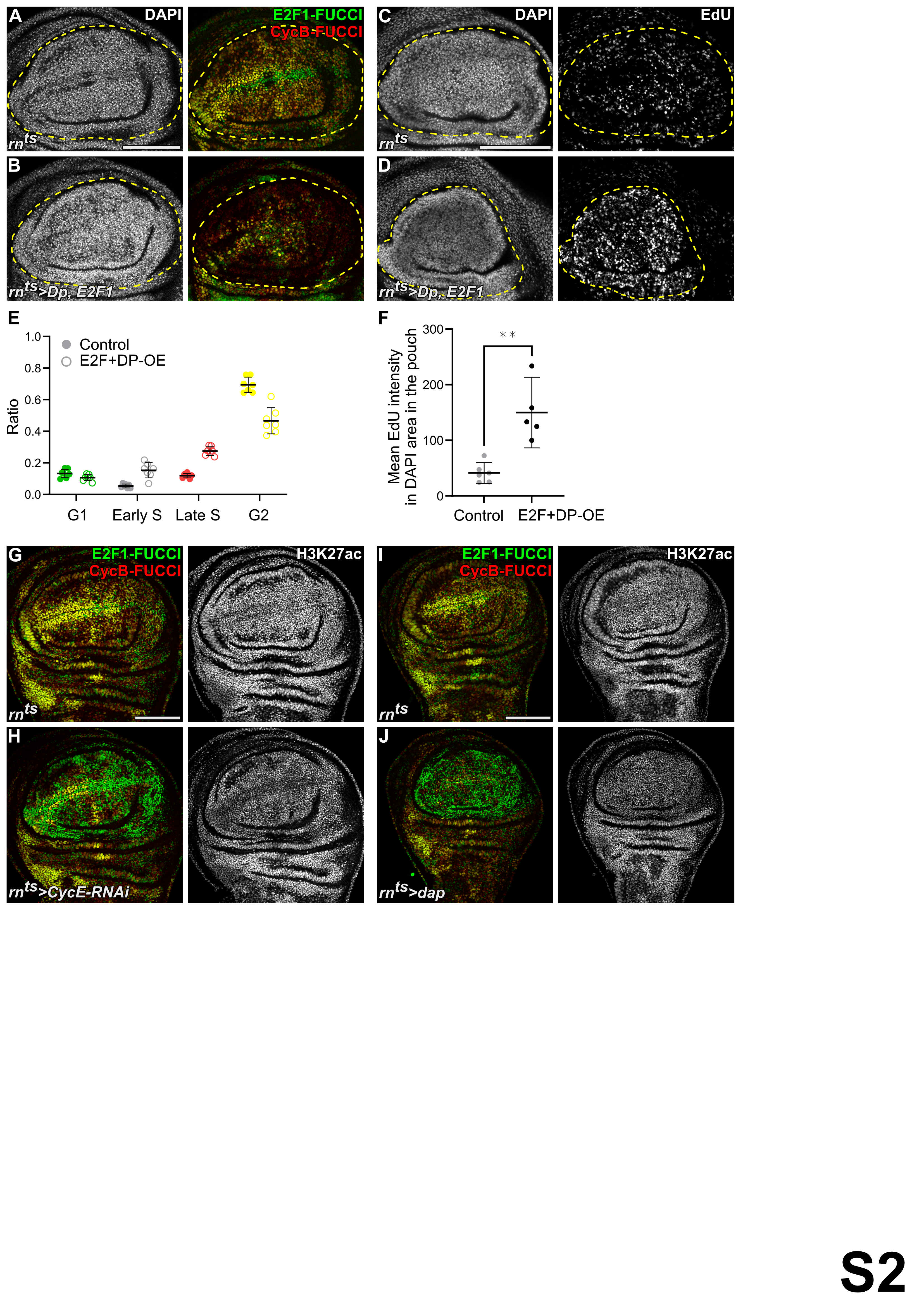

Supplement: S2 Fig — A–D. Cell cycle dynamics in control discs (A, C) and discs expressing Dp, E2F1 (B, D) for 24 h under the control of rn-GAL4. FUCCI reporters GFP-E2F11-230 (green) and mRFP-NLS-CycB1-266 (red) were used to visualize cell cycle phases (A, B) and EdU incorporation was used to detect DNA replication in S-phase cells (C, D). dDp and dE2F1 co-expression in the wing pouch domain reduces the number of cells in either G1 or G2 (E), and an increase in S-phase cells as well as an elevation of EdU incorporation (E and F) in the wing pouch region, confirming frequent entry and rapid progression through the cell cycle. E. Quantified cell cycle phase ratios in control and Dp, E2F1-coexpressing discs. Each dot represents one disc; symbols/colors denote the indicated genotypes and cell cycle phases. Error bars indicate mean ± SD. F. Quantification of mean EdU intensity per DAPI area in the pouch region of control and Dp, E2F1-coexpressing discs, serving as a proxy for relative DNA replication activity. Mean and 95% CI is shown. Statistical significance was tested using two-tailed Welch’s t test (control discs: n = 6, Dp, E2F1-expressing discs: n = 5). G-J. Cell cycle dynamics in control discs (G and I) and discs expressing CyclinE-RNAi (H) or dacapo (J) for 24 h under the control of rn-GAL4. Cell cycle phases were visualized using the FUCCI reporters GFP-E2F11-230 (green) and mRFP-NLS-CycB1-266 (red) (G-J). H3K27ac immunostaining was used to asses cell cycle-dependent changes in histone modifications. CyclinE knockdown and dacapo expression in the wing pouch shift cells toward G1 phase (H and J), and are accompanied by reduced H3K27ac levels. Discs were stained with DAPI to visualize nuclei. Fluorescence intensities are reported as arbitrary units. Yellow dashed lines represent the pouch region of wing discs. Sum projections of multiple confocal sections are shown in A-B, C-D, G-H and I-J. Scale bars: 100 μm. See S1 File for underlying data and statistical information. (TIFF) [file pbio.3003371.s004.tiff]

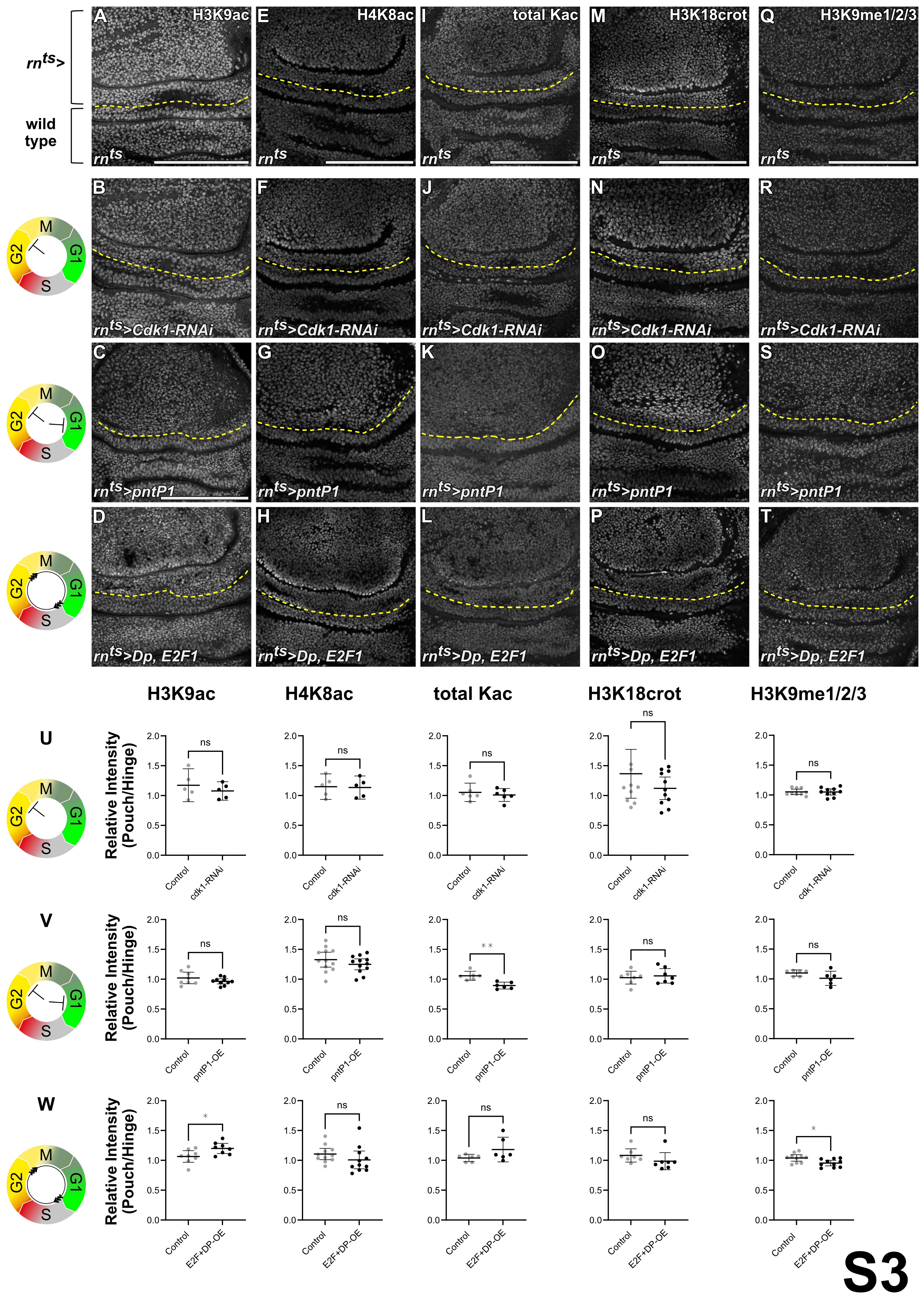

Supplement: S3 Fig — A–D. Immunostaining for H3K9ac in control (A), Cdk1-RNAi-expressing (B), pntP1-expressing (C) and Dp, E2F1-expressing (D) discs. Expression was induced for 24 h in the wing pouch using rn-GAL4. The rn-GAL4 expression pouch domain is represented by the tissue above the yellow line (rnts>). The tissue below the yellow line represents wild-type tissues of the hinge. Icons represent the experimentally verified FUCCI cell cycle status (see S1 and S2 Fig) in each condition: G2-phase arrest in Cdk1-RNAi-expressing discs (B), combined G1 and G2-phase arrest in pntP1-expressing discs (C), and cell cycle acceleration in Dp, E2F1-coexpressing discs (D). E-H. Immunostaining for H4K8ac in control (E), Cdk1-RNAi-expressing (F), pntP1-expressing (G) and Dp, E2F1-coexpressing (H) discs. I-L. Immunostaining for total acetylated lysine in control (I), Cdk1-RNAi-expressing (J), pntP1-expressing (K) and Dp, E2F1-coexpressing (L) discs. M-P. Immunostaining for H3K18crotonylation in control (M), Cdk1-RNAi-expressing (N), pntP1-expressing (O) and Dp, E2F1-coexpressing (P) discs. Q-T. Immunostaining for total H3K9me1/2/3 methylation in control (Q), Cdk1-RNAi-expressing (R), pntP1-expressing (S) and Dp, E2F1-coexpressing (T) discs. U. Quantification of relative signal intensities for H3K9ac, H4K8ac, total acetylated lysine, H3K18crotonylation, and H3K9me1/2/3, presented as pouch-to-hinge ratios (rnts-to-wild-type-cell ratio) in Cdk1-RNAi-expressing discs. Mean and 95% CI is shown. Statistical significance was tested using two-tailed Unpaired t test for H3K9ac (control discs: n = 5, Cdk1-RNAi-expressing discs: n = 5); two-tailed Unpaired t test for H4K8ac (control discs: n = 5, Cdk1-RNAi-expressing discs: n = 5); two-tailed Unpaired t test for total acetylated lysine (control discs: n = 6, Cdk1-RNAi-expressing discs: n = 6); two-tailed Mann–Whitney test for H3K18crot (control discs: n = 11, Cdk1-RNAi-expressing discs: n = 11); two-tailed Unpaired t test for H3K9me1/2/3 (control discs: n = 9, [file pbio.3003371.s005.tiff]

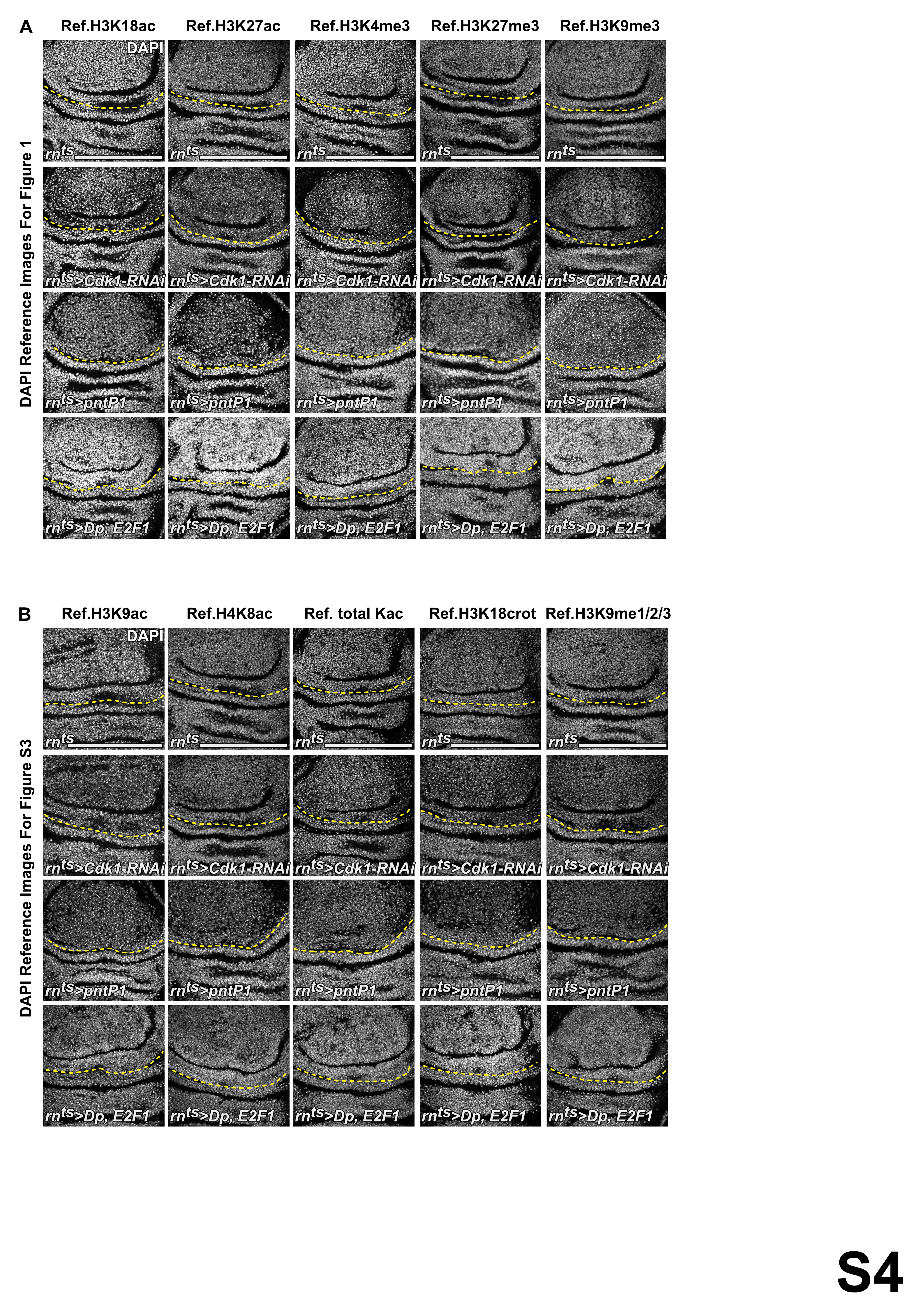

Supplement: S4 Fig — Representation of DAPI images matched to the images of histone modifications in control, Cdk1-RNAi-expressing, pntP1-expressing and Dp, E2F1-coexpressing wing discs shown in Figs 1 and S3. Discs were stained with DAPI to visualize nuclei. Sum and max projections of multiple confocal sections are shown. Scale bars: 100 μm. (TIFF) [file pbio.3003371.s006.tiff]

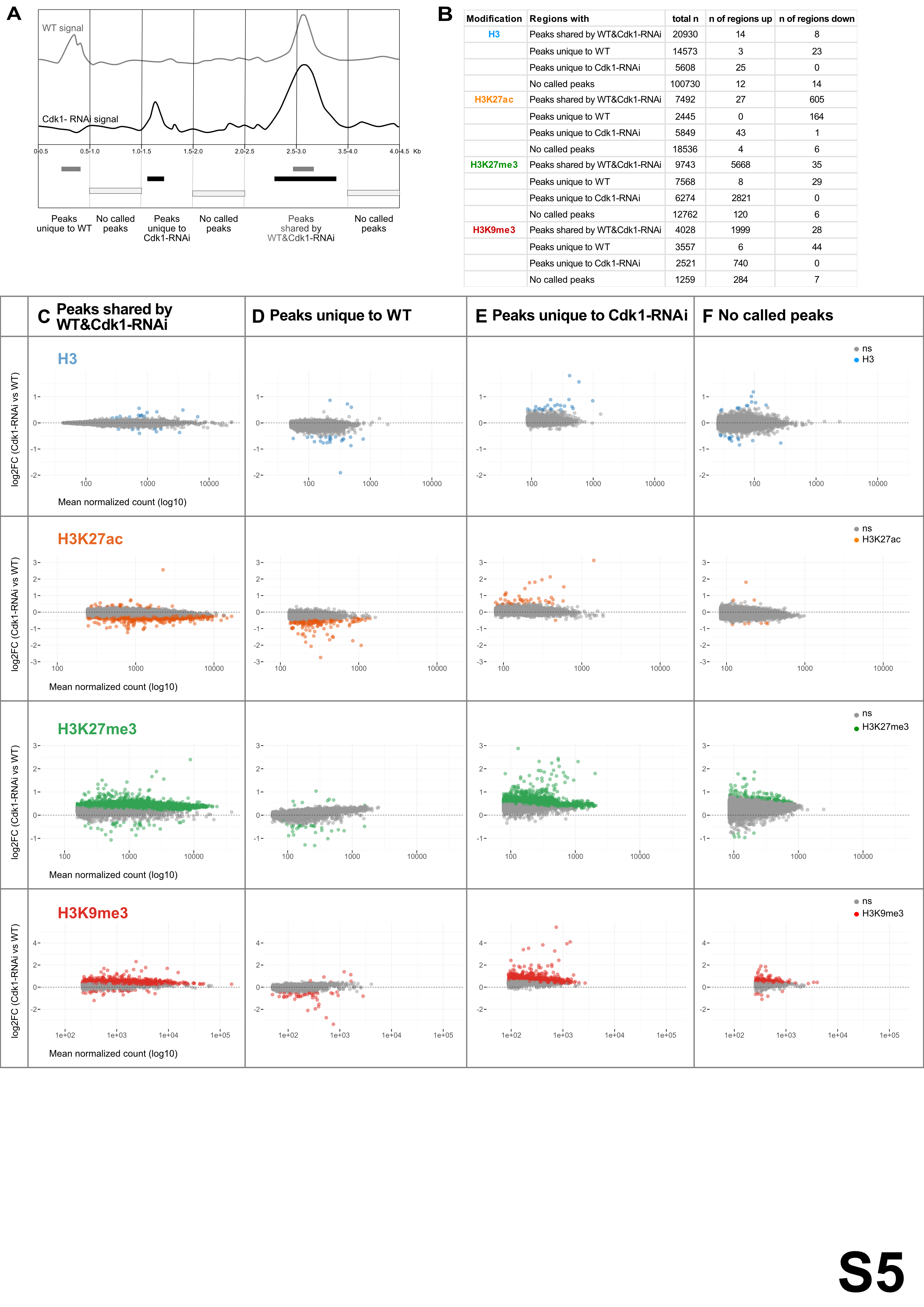

Supplement: S5 Fig — A. Schematic illustrating the genomic region categories used in the analysis. Each genomic region was categorized as containing peaks shared between WT and Cdk1-RNAi, peaks unique to WT, peaks unique to Cdk1-RNAi, or a 500-bp region with no called peaks in either condition. B. Table summarizes the total number of genomic regions described in (A) tested for each histone modification (H3, H3K27ac, H3K27me3, and H3K9me3) and the number of these regions that are significantly upregulated (log2FoldChange> 0) or significantly downregulated (log2FoldChange < 0) in Cdk1-RNAi relative to WT. C-F. MA plots displaying differential enrichment of histone modifications measured by CUT&Tag for H3, H3K27ac, H3K27me3, and H3K9me3 (rows). Each point represents a genomic region classified by peak category as defined in our analysis: regions with peaks shared between WT and Cdk1-RNAi (C), peaks unique to WT (D), peaks unique to Cdk1-RNAi (E), or 500-bp regions with no called peaks in either condition (F). The x-axis shows the mean normalized read count (log10), and the y-axis shows the log2 fold change (Cdk1-RNAi/WT). Points are colored by histone modifications (H3, blue; H3K27ac, orange; H3K27me3, green; H3K9me3, red). Regions with DESeq2 adjusted P < 0.1 are shown in color, whereas non-significant regions (ns; adjusted P ≥ 0.1) are shown in gray. Access to underlying data is provided at NCBI (SRA) PRJNA130380. See Code and Data Availability Statement. (TIFF) [file pbio.3003371.s007.tiff]

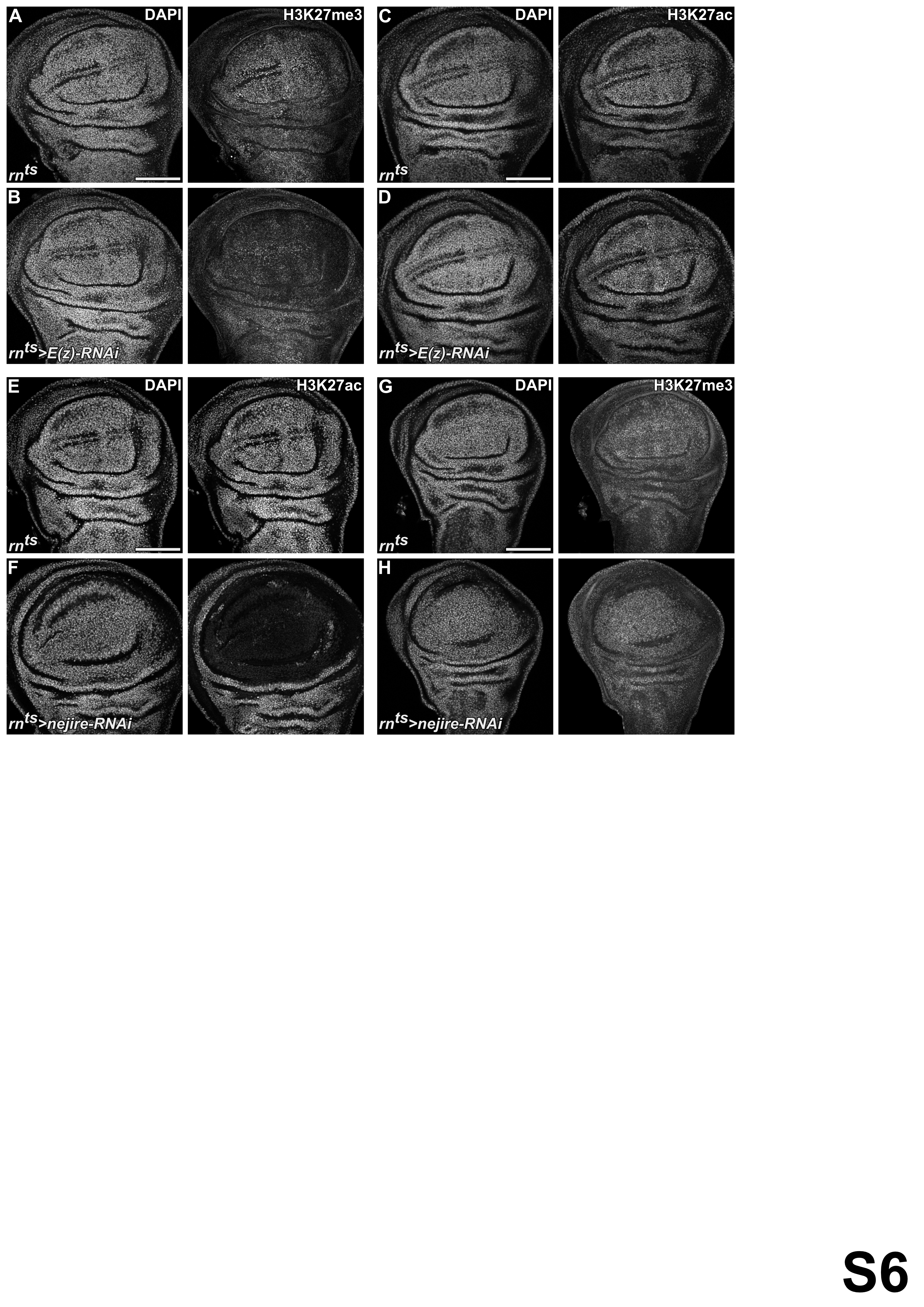

Supplement: S6 Fig — A-B. H3K27me3 staining in control (A) or E(z)-RNAi-expressing (B) discs. Knockdown of the H3K27me3 writer E(z) causes a reduction in H3K27me3 level in the pouch of the wing disc. C-D. H3K27ac staining in control (C) or E(z)-RNAi-expressing (D) discs. Knockdown of the H3K27me writer E(z) causes no corresponding increase in H3K27ac level. E-F. H3K27ac staining in control (E) and nejire-RNAi-expressing (F) discs. Knockdown of the H3K27ac writer CBP/nej causes a reduction of H3K27ac level in the pouch of the wing disc. G-H. H3K27me3 staining in control (G) and nejire-RNAi-expressing (H) discs. Knockdown of the H3K27ac writer CBP/nej causes no corresponding increase in H3K27me3 level. Discs were stained with DAPI to visualize nuclei. Maximum projections of multiple confocal sections are shown in A-B. Sum projections of multiple confocal sections are shown in C-D, E-F and G-H. Scale bars: 100 μm. (TIFF) [file pbio.3003371.s008.tiff]

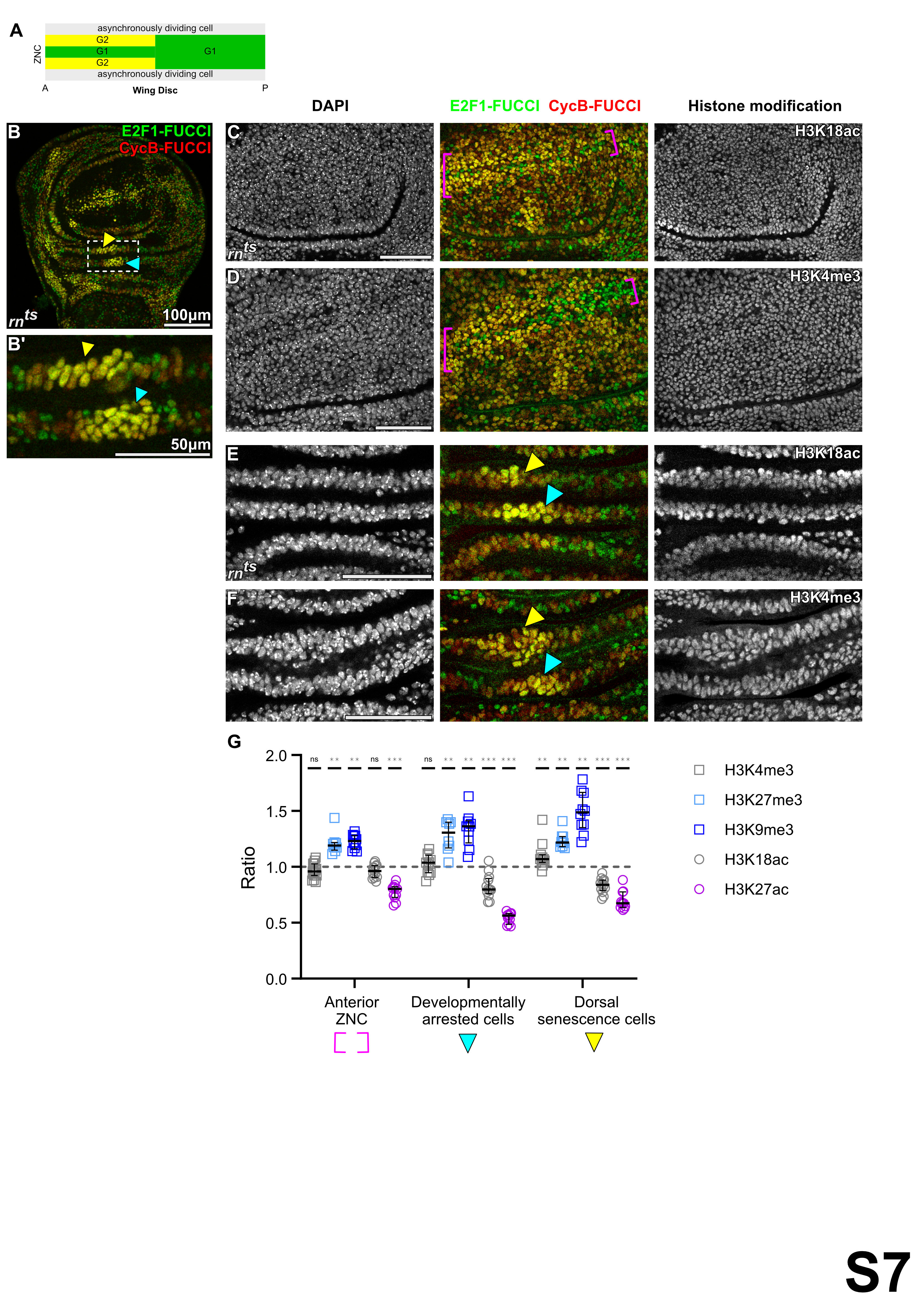

Supplement: S7 Fig — A. Schematic representation of the zone of nonproliferation cells (ZNC) (adapted from [72]). B-B’. The Fly-FUCCI system was used to visualize the spatial pattern of developmentally arrested cells (cyan arrowhead) and dorsal senescence cells (yellow arrowhead) in hinge region of normally developing wing disc. C-D. Immunostaining for H3K18ac (C) and H3K4me3 (D) in the zone of nonproliferation cells (ZNC) of developing wing discs. Neither histone modification shows detectable changes in this region. E-F. Immunostaining for H3K18ac (E) and H3K4me3 (F) in the wing disc hinge region; programmed senescence is indicated by yellow arrowheads and developmental cell cycle arrest is indicated by cyan arrowheads. G. Quantification of ratios of histone modification levels within the anterior ZNC, developmentally arrested cells, and dorsal senescence cells relative to regions outside each domain (see Materials and Methods for details) in normally developing wing discs. For each condition, a one-sample Wilcoxon signed-rank test was used to test whether the median ratio differed from 1. Symbols represent individual wing discs; horizontal black lines indicate the median and error bars indicate the IQR. For H3K4me3; anterior ZNC n = 15, developmentally arrested cells n = 11, dorsal senescence cells n = 11. For H3K27me3; anterior ZNC n = 8, developmentally arrested cells n = 8, dorsal senescence cells n = 8. For H3K9me3; anterior ZNC n = 10, developmentally arrested cells n = 10 and dorsal senescence cells n = 10. For H3K18ac; anterior ZNC n = 13, developmentally arrested cells n = 13 and dorsal senescence cells n = 13. For H3K27ac; anterior ZNC n = 11, developmentally arrested cells n = 11 and dorsal senescence cells n = 11. Discs were stained with DAPI to visualize nuclei. Fluorescence intensities are reported as arbitrary units. Sum projections of multiple confocal sections are shown in B-B’. Scale bars: 100 μm in B; 50 μm in B’ and C-H. See S1 File for underlying data and statistic [file pbio.3003371.s009.tiff]

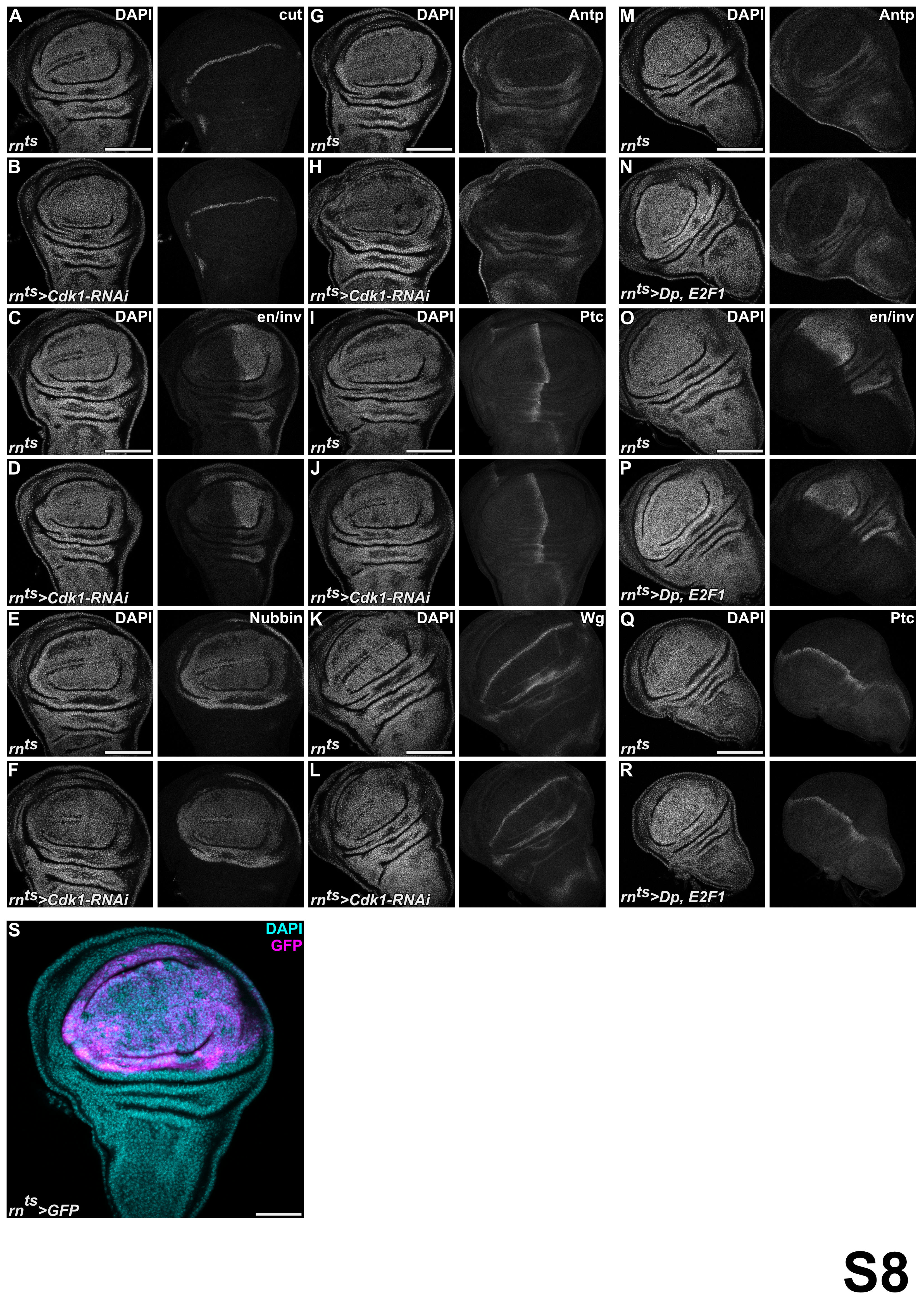

Supplement: S8 Fig — A-L. Immunostaining for proteins expressed by Polycomb target genes: Cut (A, B), Engrailed/invected (C, D), Nubbin (E, F), Antennapedia (G, H), Patched (I, J) and Wingless (K, L) in control (A, C, E, G, I, K) and Cdk1-RNAi-expressing (B, D, F, H, J, L) wing discs. M-R. Immunostaining for proteins expressed by Polycomb target genes: Antennapedia (M, N), Engrailed/invected (O, P) and Patched (Q, R) in control (M, O, Q) and Dp, E2F1-coexpressing (N, P, R) wing discs. S. A control wing disc after 24 h of UAS-GFP-expression in the pouch (magenta), under the control of the rn-GAL4 (rotund-GAL4) driver, displayed to reference the manipulated pouch domain in Figs A-R. Please note that this is the same disc shown in S1A Fig. Discs were stained with DAPI to visualize nuclei. Sum projections of multiple confocal sections are shown in A-S. Scale bars: 100 μm. (TIFF) [file pbio.3003371.s010.tiff]

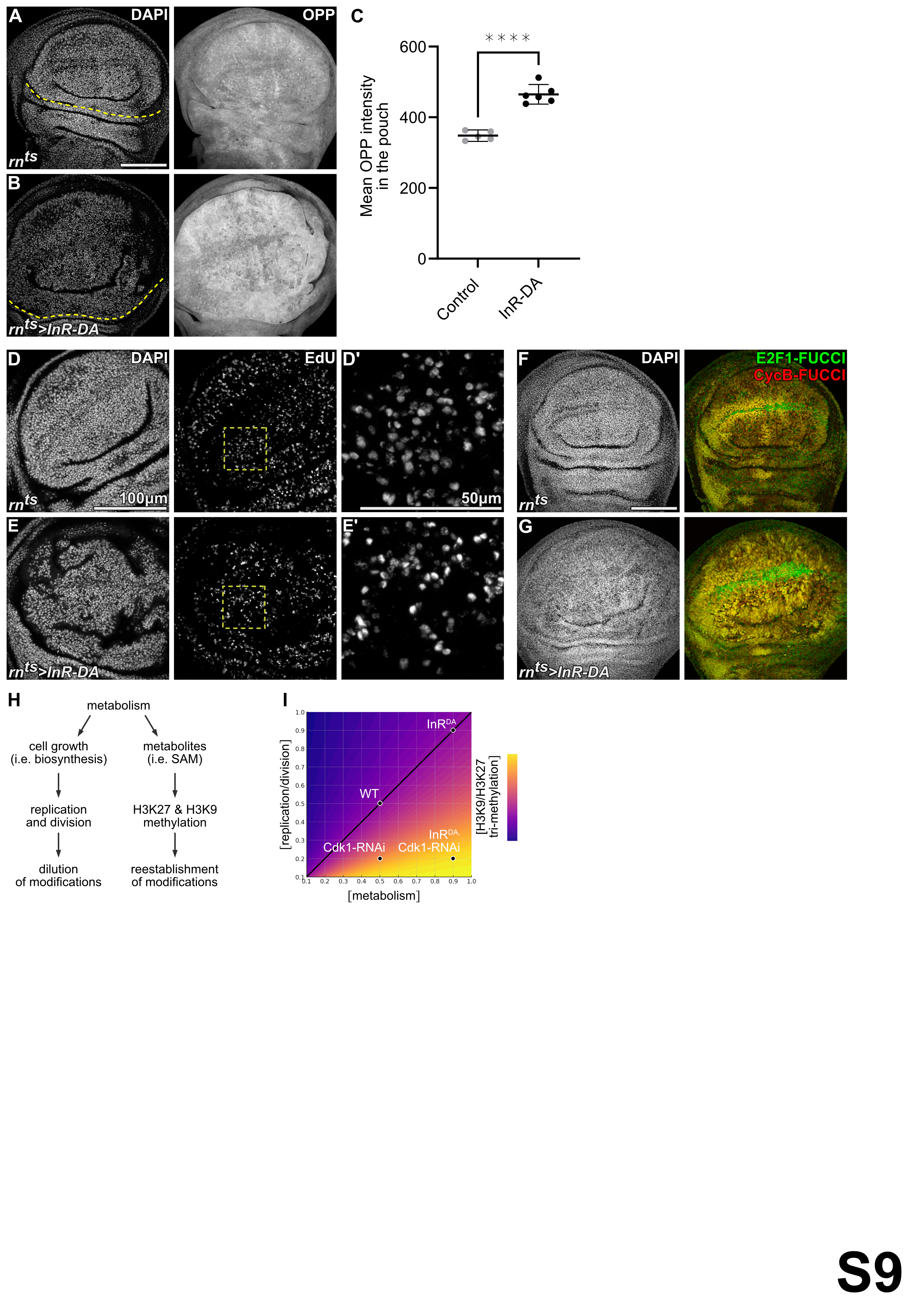

Supplement: S9 Fig — A-B. Protein synthesis visualized by OPP incorporation in control (A) and InR-DA-expressing (B) discs. Yellow dashed lines indicate the boundary between the wing pouch and hinge regions. The area above the line corresponds to the rn-GAL4 expression domain, while the area below represents wild-type cells. The InR-DA-expressing wing pouch exhibits higher rates of protein synthesis, reflecting an increased biosynthetic capacity promoted by higher InR/PI3K/Akt activity. C. Quantification of mean OPP intensity in the pouch region of control and InR-DA-expressing discs. Mean and 95% CI is shown. Statistical significance was tested using two-tailed Unpaired t test (control discs: n = 5, InR-DA-expressing discs: n = 6). D-E. EdU incorporation visualizes DNA replication in the pouch region of control (D) and InR-DA-expressing (E) discs. Dashed yellow squares highlight the magnified regions shown in (D’ and E’). InR-DA-expressing wing pouch shows higher levels of EdU intensities, reflecting accelerated speed of DNA replication. Rates of EdU incorporation in InR-DA-expressing discs were previously reported and quantified [63]. F-G. Cell cycle dynamics in control (F) and InR-DA-expressing (G) discs. FUCCI reporters GFP-E2F11-230 (green) and mRFP-NLS-CycB1-266 (red) were used to visualize cell cycle phases. Note that expression of a constitutively active Insulin receptor does not alter the cell cycle phase profile of the wing pouch compared to control disc. H. Illustration of the dual role of metabolism in regulating H3K9 and H3K27 trimethylation levels. Metabolism contributes to the dilution of histone PTMs by accelerating the cell cycle, thereby increasing the frequency of DNA replication events. Simultaneously, it supports the reestablishment of these PTMs by supplying essential precursors and cofactors required for methyltransferase activity. I. Hypothetical model depicting the relationship between histone H3K9 and H3K27 trimethylation levels, metabolic rate, and cell divisi [file pbio.3003371.s011.tiff]

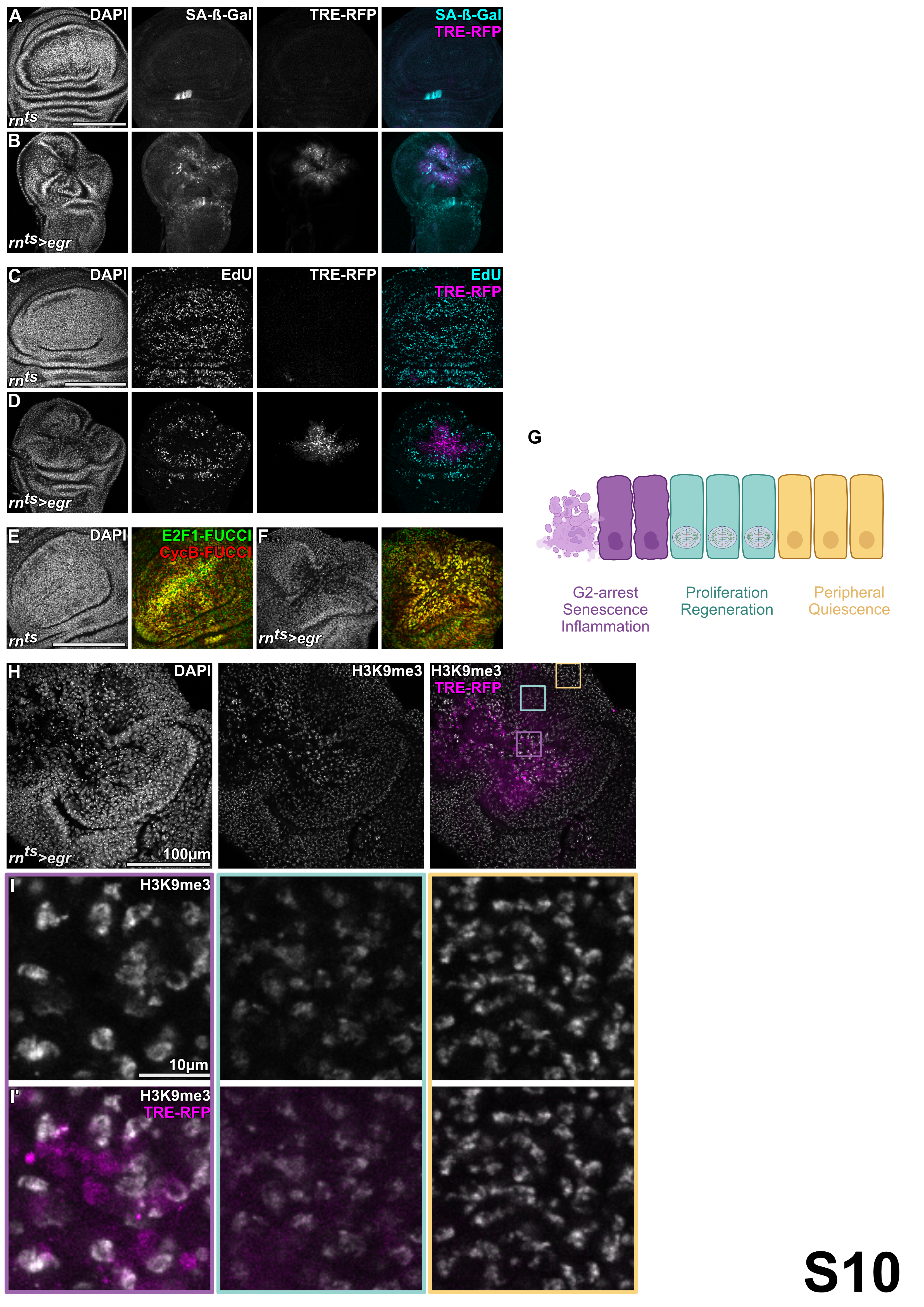

Supplement: S10 Fig — A-B. Senescence-associated β-galactosidase (SA-β-gal) activity (cyan or gray) in control (A) and egr-expressing (B) discs. TRE-RFP reporter (magenta) visualizes JNK-pathway activity. SA-β-gal activity in egr-expressing discs was previously reported and quantified [77]. C-D. EdU incorporation (gray or cyan) visualizes DNA replication in control (C) and egr-expressing (D) discs. TRE-RFP reporter (magenta) visualizes JNK-pathway activity. EdU incorporation was previously reported and quantified [77,76]. E-F. Cell cycle dynamics in control (E) and egr-expressing (F) discs. FUCCI reporters GFP-E2F11-230 (green) and mRFP-NLS-CycB1-266 (red) were used to visualize cell cycle phases. G. A model illustrating inflammatory tissue damage in Drosophila wing imaginal discs. Eiger expression activates the JNK pathway, an early stress response, which induces a senescence-like cell cycle arrest in the G2 phase at center of the tissue damage and triggers a senescence program [64]. These arrested cells secrete Unpaired cytokines, which activate the JAK/STAT pathway in surrounding cells, promoting compensatory proliferation during tissue regeneration [63,76,77,111–114]. At the periphery of the damage site, cells remain in a quiescent state [115]. H-I’. Immunostaining for H3K9me3 in egr-expressing disc (H). The magenta frame highlights G2-arrested senescent cells, which show elevated H3K9me3 levels. The cyan frame marks cells undergoing compensatory proliferation, where H3K9me3 levels are reduced. The yellow frame indicates quiescent cells, which have higher H3K9me3 levels than proliferating cells (I). TRE-RFP reporter signal (magenta) is elevated in G2-arrested senescent cells but is absent in cells undergoing compensatory proliferation or quiescence (I’). Discs were stained with DAPI to visualize nuclei. Maximum projections of multiple confocal sections are shown in H. Sum projections of multiple confocal sections are shown in A-B, C-D and E-F. Scale bars: 100 μm in A-B, C-D, E-F and [file pbio.3003371.s012.tiff]

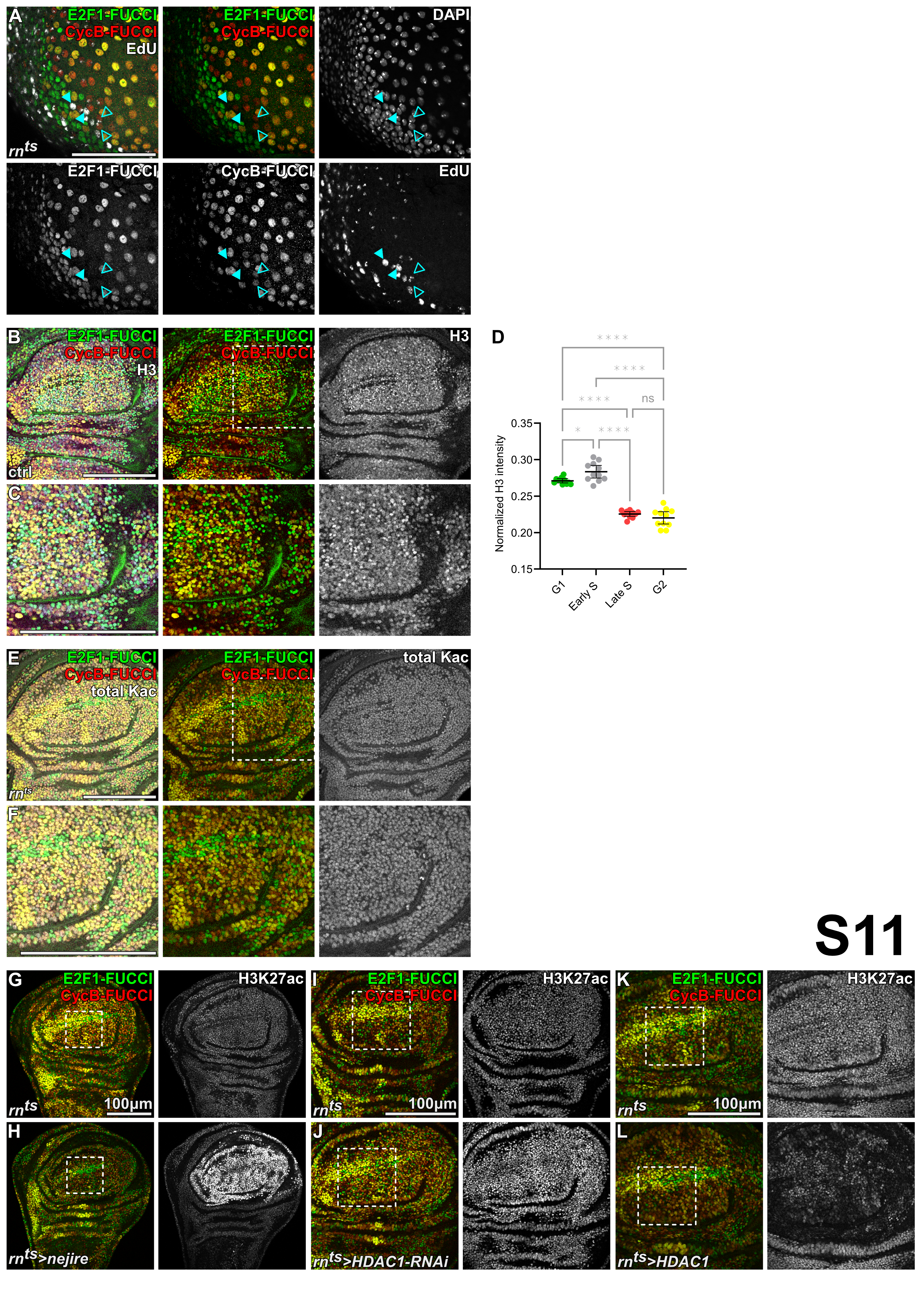

Supplement: S11 Fig — A. FUCCI reporter and EdU incorporation assays in the peripodium of a wild-type wing disc. Euchromatic EdU incorporation (early S-phase) correlates with absence of fluorescence from both FUCCI reporters (GFP-E2F11-230 (green) and mRFP-NLS-CycB1-266 (red); filled arrowheads). Heterochromatic EdU incorporation (late S-phase) correlates with a modest increase in the G2-specific reporter mRFP-NLS-CycB1-266 (red; open arrowheads). Cells with elevated levels of both FUCCI reporters (yellow) are in late G2 [66]. B-C. Immunostaining for H3 in a developing control disc (B), with a magnified region (demarcated by a white dashed square) shown in panel C, also expressing the FUCCI reporter (GFP-E2F11-230 (green) and mRFP-NLS-CycB1-266 (red)) to visualize cell cycle phases. D. Quantification of normalized H3 intensity level with respect to different cell cycle phases in the pouch region of control discs. Normalization was performed to the average fluorescence intensity across all FUCCI-defined phases (to avoid privileging any single phase as the normalization reference). Mean and 95% CI is shown. Statistical significance was tested using Repeated Measures One-Way ANOVA followed by Tukey’s post-hoc test for multiple comparison (n = 11 discs). E-F. Immunostaining for total acetylated lysine level in a developing control disc (E), with a magnified region (demarcated by a white dashed square) in panel F, also expressing the FUCCI reporters (GFP-E2F11-230 (green) and mRFP-NLS-CycB1-266 (red)) to visualize cell cycle phases. G-H. Immunostaining for H3K27ac in control (G) and nejire-expressing (H) discs with FUCCI reporters (GFP-E2F11-230 (green) and mRFP-NLS-CycB1-266 (red)) to visualize cell cycle phases. The location of magnified regions shown in Main Fig 5I and 5J is indicated by white dashed squares. I-J. Immunostaining for H3K27ac in control (I) and HDAC1-RNAi-expressing (J) discs with FUCCI reporters (GFP-E2F11-230 (green) and mRFP-NLS-CycB1-266 (red)) to visualize cell cycle ph [file pbio.3003371.s013.tiff]

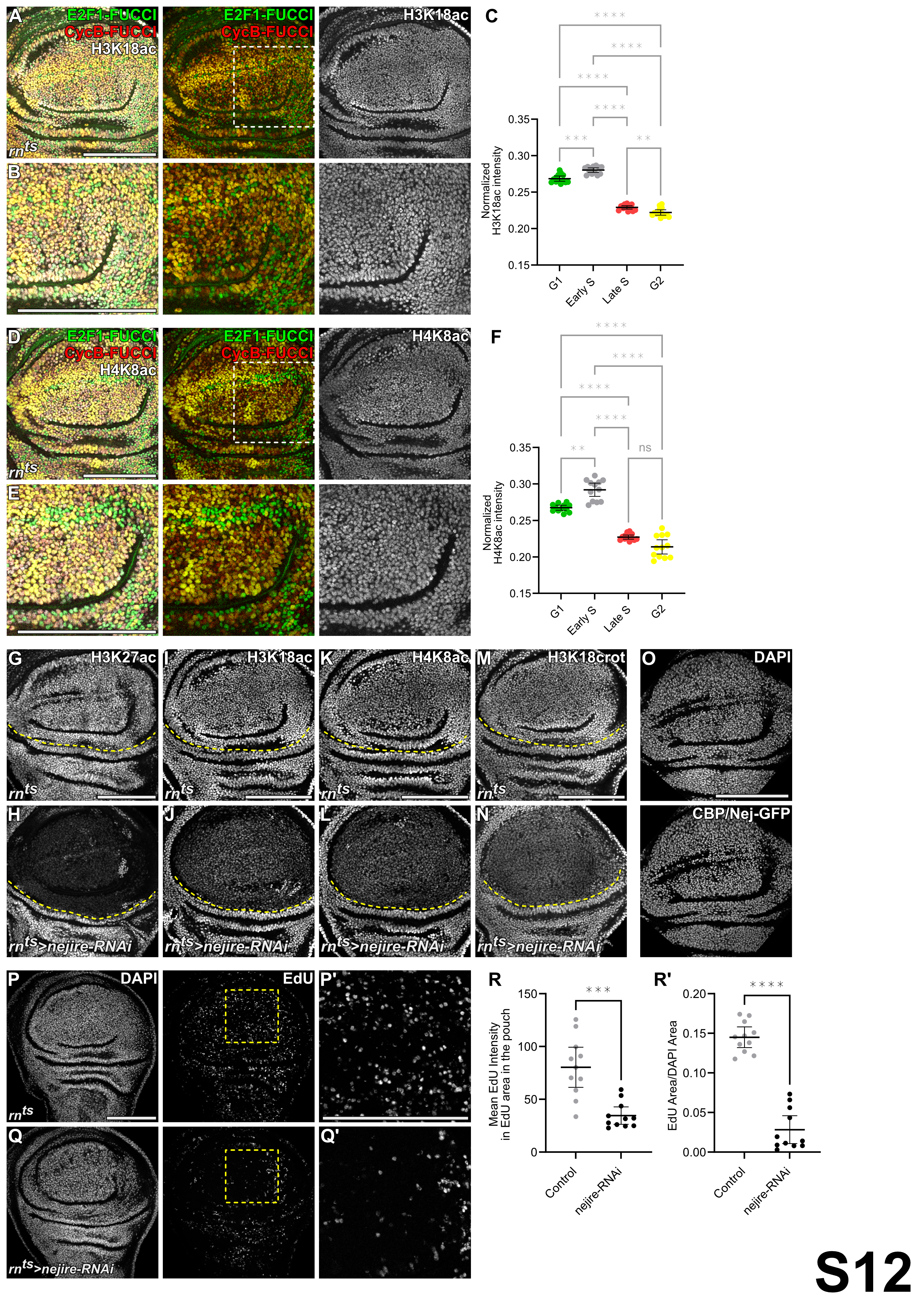

Supplement: S12 Fig — A-B. Immunostaining for H3K18ac in a developing control disc (A), with a magnified region (demarcated by a white dashed square) in panel B, in the background of FUCCI reporters (GFP-E2F11-230 (green) and mRFP-NLS-CycB1-266 (red)) to visualize cell cycle phases. C. Quantification of normalized H3K18ac intensity level in different cell cycle phases in the pouch region of control discs. Normalization was performed to the average fluorescence intensity across all FUCCI-defined phases (to avoid privileging any single phase as the normalization reference). Mean and 95% CI is shown. Statistical significance was tested using Repeated Measures One-Way ANOVA followed by Tukey’s post-hoc test for multiple comparison (n = 13 discs). D-E. Immunostaining for H4K8ac in a normally developing control disc (D), with a magnified region (demarcated by a white dashed square) in panel E, also expressing the FUCCI reporters (GFP-E2F11-230 (green) and mRFP-NLS-CycB1-266 (red)) to visualize cell cycle phases. F. Quantification of normalized H4K8ac intensity level in different cell cycle phases in the pouch region of control discs. Normalization was performed to the average fluorescence intensity across all FUCCI-defined phases (to avoid privileging any single phase as the normalization reference). Mean and 95% CI is shown. Statistical significance was tested using Repeated Measures One-Way ANOVA followed by Tukey’s post-hoc test for multiple comparison (n = 12 discs). G-H. Immunostaining for H3K27ac in control (G) and nejire-RNAi-expressing (H) discs. Same disc is also shown in Fig 5E and 5F but repeated here to allow for direct comparison with (I-N). I-J. Immunostaining for H3K18ac in control (I) and nejire-RNAi-expressing (J) discs. K-L. Immunostaining for H4K8ac in control (K) and nejire-RNAi-expressing (L) discs. M-N. Immunostaining for H3K18crot in control (M) and nejire-RNAi-expressing (N) discs. Yellow dashed lines indicate the boundary between the wing pouch and hinge regions. O. Ex [file pbio.3003371.s014.tiff]
