# Supplementary material for: Cell cycle dynamics regulate H3K27 and H3K9 histone modifications in Drosophila
Source: PLoS Biol. 2026 Mar 26;24(3):e3003371. doi: 10.1371/journal.pbio.3003371 (PMC13046271; doi:10.1371/journal.pbio.3003371)
Supplement: Attachment — Submitted filename: 260128 Response to reviewer .pdf [file pbio.3003371.s017.pdf]

Comments from the Reviewers

Response to Reviewer 1-4

We are grateful to the Reviewers and Editor for their constructive feedback, which has helped us substantially improve the manuscript. We have revised the manuscript extensively to address the concerns raised and sharpened the focus of our message.

Specifically, in response to concerns raised by all reviewers:

- We added new measurements, quantifications, and statistical analyses in all main figures and the supplement to ensure that all key conclusions are supported quantitatively. We provide a new Source data file that compiles the underlying data points, replicate numbers, and statistical tests for all quantifications.

In parallel, we focused the narrative:

- In line with editorial guidance, we removed the former Figure 6 and associated claims that CBP/Nejire-mediated H3K27ac directly prevents replication stress/DNA damage. We agree with the reviewers that indirect mechanisms (e.g., transcriptional effects, replication–transcription conflicts) cannot be excluded.

- To provide better mechanistic insights, we expanded Figure 5 and included new experimental data to resolve the residue-specific, cell-cycle-structured H3K27ac dynamic in vivo. By directly juxtaposing H3K27ac, total H3 and total acetylation dynamics across FUCCI-defined cell cycle phases, we show that H3K27ac peaks sharply in early S phase and remains elevated into late S phase. Importantly, we demonstrate that cell cycle phase program is strikingly robust and depends on the enzyme balance of the Nejire writer and HDAC1 eraser. Together, these data establish H3K27ac dynamics as a regulated, S-phase-resolved feature of chromatin state in a developing tissue in vivo.

We provide a detailed point-by-point response below and indicate where changes were made in the revised manuscript.

For easier access to the changes, we provide the following details:

New data, quantifications, graphs and tables can be found in these positions:

- Figure 2 F
- Figure 3 J
- Figure 4 P, Q, R
- Figure 5 M, N, O’, P’, Q, Q’
- Figure S1.1 G, M
- Figure S1.2 E, G, H, I, J
- Figure S1.4
- Figure S2.1 A, B
- Figure S3.1 B, B’, G
- Figure S4.2 H (TRE-RFP channel was added)
- Figure S5.1 A, K, L

- New ‘Source data’ file with quantification data points, replicates and statistics

The following figures have been relocated:

- Old Figure S1 G to New Figure S1 H
- Old Figure S1 H, I, J, K to New Figure S1 I, J, K, L
- Old Figure S1 L to New Figure S1.N
- Old Figure S1 M, N, O, P, Q to New Figure S1.2 A, B, C, D, F
- The old Figure S1.2 now became S1.3 and old S1.3 became S1.4. Also, we divided the S1.1 into two figures; S1.1 and S1.2 to fit the data blocks with their quantifications together.
- Old Figure 2 A to New Figure 2 G
- Old Figure 2 B to New Figure S2.1 G
- Old Figure 2 C, D, E, F to New Figure 2 A, B, C, D
- Old Figure S2.1 A, B, C, D to New Figure S2.1 C, D, E, F
- Old Figure S2.1 E to New Figure 2 E
- Old Figure 3 A, B, C, D, E, F to New Figure 3 D, E, F, G, H, I
- Old Figure S3.1 B, C, D to New Figure 3 A, B, C
- Old Figure S3.1 E, F, G, H to New Figure S3.1 C, D, E, F
- Old Figure 4 P, Q to New Figure S4.1 H, I
- Old Figure 5 D, E to New Figure 5 E, F
- Old Figure 5 F, G, H, I to New Figure 5 I, J, K, L
- Old Figure 5 J, K to New Figure 5 O, P
- Old Figure 6 I, J to New Figure 5 G, H
- Old Figure S5.1 A, B, C, D, E to New Figure S5.1 B, C, D, E, F
- Old Figure S5.1 F to New Figure 5 D
- Old Figure 6 A, B, C, C’ to New Figure S5.2 P, P’, Q, Q’, R, R’

The following figures were removed:

- Old Figure 6 D, E, F, G, H, K, L
- Old Figure S6

Reviewer #1:

The manuscript by Nogay and coworkers addresses the interplay between cell cycle progression and histone modifications, which is an interesting and understudied area in the field of epigenetics. As a model system they use the Drosophila wing imaginal disc, which to my knowledge is the first attempt to study this in an in vivo system. By manipulating the cell cycle, the authors identify changes in H3K27me3, H3K9me3 and H3K27ac. They find that H3K27me3 and H3K9me3 accumulate in cells arrested in either G2 or G1, whereas E2F over-expression which should speed up the cell cycle leads to a reduction in these marks. H3K27ac show the opposite behavior.

They show that a physiological change of cell cycle speed would not lead to a change in H3K27me3 and H3K9me3 levels (what about H3K27ac?), and provide an attractive hypothesis that a balanced increase in proliferation and metabolic activity ensures that enzymatic activity matches the shorter cycling time.

They study H3K27ac separately and show that the levels are elevated in early S phase. They observed increased DNA damage upon depletion of the HAT Nejire and in a H3K27R line, and propose that H3K27ac directly counteracts replication stress.
The study is interesting and broadly relevant to the chromatin and cell cycle fields, in particular with respect to the findings on H3K27me3 and H3K9me3 which are exciting. However, the conclusions about the function of H3K27ac in replication stress protection are not convincingly supported by the data.

Concerns
1. The interpretation of the data on H3K27ac in Figure 5 and 6 is problematic.
Fig 5:
The authors first conclude that K27ac is higher in early and late S phase. Yet compared to G1, only early S phase is higher in K27ac. It is not advisable to compare to G2, as it is well established that the epigenome is deacetylated prior to entry into mitosis. Therefor the data suggest that early S phase cells are higher in K27ac, not S phase in general as stated.

Indeed, based on the quantifications, the most direct conclusion is that H3K27ac is highest in early S phase compared with other FUCCI-defined phases. At the same time, we observed an additional dynamic when comparing H3K27ac to bulk histone acetylation dynamics. Bulk acetylation sharply peaks in early S and then declines, whereas H3K27ac remains elevated in late S compared to bulk acetylation, suggesting that H3K27 acetylation may follow a residue-specific program rather than that of global acetylation. To improve the clarity of this conclusion, we now present bulk acetylation and H3K27ac side-by-side in Fig. 5 and directly compare global and H3K27ac trajectories.

Regarding the concern about reduced acetylation in G2 due to pre-mitotic deacetylation: we agree that inclusion of pre-mitotic cells could then confound G2 measurements. In our quantifications, we thus always excluded apically positioned pre-mitotic and mitotic cells, so that our analysis only includes bulk G2 phase cells (see manuscript line 330 ff). To address this concern even more directly we also stratified the G2 cells which we include in our analysis into early and late G2 cells based on increasing levels of FUCCI-red intensities. When doing that, we do not detect a drop in H3K27ac between early and late G2 cells included in our analysis (see graph below).
In addition, we confirmed that we also see a downregulation of H3K27ac in G1-arrested cells suggesting that loss of H3K27ac is not just a feature of G2 arrested cells (see Fig S1.2 G-J).

Of note: Please consider that in Fig. 5 we did not normalize H3K27ac intensities to G1! Instead, for each disc we normalized to the mean H3K27ac signal across all FUCCI-defined phases (i.e., the global average), because this makes the relative differences between phases easier to compare without privileging any single phase as the reference.

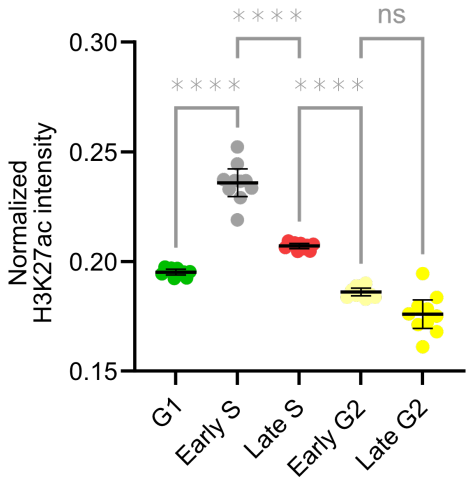

The rational from the over-expression experiment is unclear. If you overexpress an enzyme, one may not expect that it maintains it normal regulation. Why is G2 and early S cells not shown? For the HDAC1 knockdown, might the effect be related to the de-acetylation happening before mitosis? What happens in early S and G2?

Especially since CBP protein levels did not fluctuate during the cell cycle, regulation of H3K27ac where likely not controlled by levels but cell cycle dependent regulation. The intent for this experiment was to ask whether or not H3K27ac levels remain coupled to cell-cycle phase even when Nejire levels are experimentally altered. We observe that H3K27ac indeed remains cell-cycle-phase correlated even when Nej (or HDAC) levels are increased or decreased, consistent with an additional layer of cell-cycle-linked control acting on H3K27ac. We have now included quantification across all FUCCI-defined phases (G1, early S, late S, and G2) for Nej overexpression, HDAC overexpression, and HDAC RNAi in the completely revised Fig. 5. (We did not include phase-resolved quantification for Nej RNAi because it causes a strong G2 arrest. The data shows that across Nej/HDAC perturbations, H3K27ac remains cell cycle phase-patterned, with a robust enrichment in S-phase. Notably, the increase in H3K27ac from G1 to early S scales with the G1 baseline: Nej overexpression and HDAC RNAi elevate H3K27ac overall, (including in G1). We find that there is now a relatively lower increase from G1 to early S-phase than in wild type cells. In contrast, HDAC overexpression lowers H3K27ac overall (again including in G1), and this is accompanied by a relatively larger increase in H3K27ac when cells transition to early S-phase. In contrast, the early S to late S transition is most sensitive to enzyme balance: Nej overexpression/HDAC RNAi cells retain higher H3K27ac into late S, while HDAC overexpression drives a stronger late-S-phase decrease. Together, these data demonstrate that H3K27ac exhibits robust cell cycle linked dynamics independent of net acetylation rates and support a model where early-S phase enrichment of H3K27ac is mediated by CBP/nej, whereas its removal in late S-phase depends on HDAC1.

Fig. 6:
The conclusion that CBP/nej is specifically required to prevent genome-wide replication stress cannot be made from the presented data. The rationale is flawed as it ignores the established role of K27ac in transcription. Firstly, they provide no direct link between the DNA damage signal and S phase/ DNA replication. Would lack of replication prevent damage? Secondly, damage could arise as a secondary effect of K27ac affecting expression of key replication/repair factors and/or replication-transcription conflicts. Thus, it cannot be concluded that the effect is direct. Direct effects on DNA replication / genome stability by

K27ac would have to be established via specific mechanisms. I therefore strongly recommend that this part is removed (Fig. 6).

We agree that, based on the data presented, we cannot exclude indirect mechanisms (transcriptional effects on replication and DNA repair factors, or replication-transcription conflicts), nor do we provide a direct link between H3K27ac-regulated DNA damage and DNA replication. Our experimental system is limited regarding approaches to pharmacological intervention of transcription or replication, and inhibition by genetic tools have profound pleiotropic phenotypes. Therefore, we have removed Figure 6 and the associated claims that CBP/Nejire-mediated H3K27ac directly prevents replication stress.

2. The cell cycle distributions are not very well documented across the manuscript. The authors use the FUCCI system, but do not quantify the changes. This should be done across the different setups (Figure 1 and 4).

We have now added quantifications of cell cycle phases for the manipulations shown in Fig. 1 and supplements. Our measurements are consistent with prior characterizations of these perturbations using FUCCI, flow cytometry, EdU or clonal analysis; for example (Neufeld et al., 1998, Buttitta et al., 2007, Bettencourt-Dias et al., 2004, Ruiz-Losada et al., 2022, Ito and Igaki, 2021, Zielke et al., 2014, Handke et al., 2014).
For Figure 4 we are technically restricted: we cannot generate a FUCCI, InR^{CA} and Cdk1-RNAi genotype because of genetic limitations (distribution of required transgenes on different chromsomes). However, the Cdk1-RNAi genotype is quantified in Fig 1 and a representative image for InR^{CA} alone is shown in supplements to Figure 4. In addition, we cite (and include below) our prior work , showing that InR^{CA} expressing disc experience more S-phase entry and faster S-phase progression (and thus faster growth) based on EdU incorporation – see Fig 8 in (Crucianelli et al., 2022).

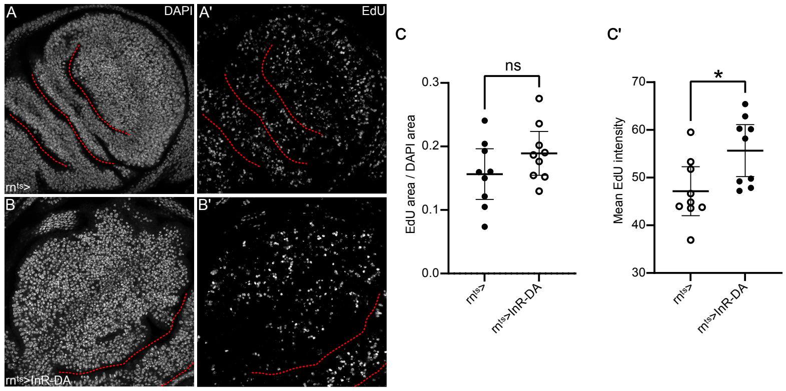

Over-expression of E2F1 is expected to shorten G1 and push cells faster into S phase, it is therefore surprising that the authors state that there are no changes in the proportion of G1 and G2 cells. It looks like there are more cells in S - which should lead to a proportional reduction in G1/G2. Quantification of the different cell cycle stages in the models should clarify this.

We agree that this statement is confusing, as Dp,E2F overexpression should bias cells toward S-phase. Our original statement was meant to indicate that the G1:G2 ratio does not change. To address your concern directly, we now provide the quantification of cell-cycle distributions for Dp,E2F overexpression in the

revised supplements to Fig 1. Indeed, we observe an increase in the proportion of S-phase cells, accompanied by a proportional reduction of both G1 and G2 populations.

3. Please comment on whether the Cut&Tag analysis is quantitative. Was spike-in applied, and, if not, what caveats should be considered.

Yes, Our CUT&Tag datasets were generated and analyzed in a quantitative manner using both H3 and exogeneous spike-in signals for normalization, which allows comparison of global signal level between conditions. We calculated the scaling factors from the normalization of the merged data and applied these scaling factors to the replicate-level data for quantitative analysis using *DESeq2-v1.38.3* pipeline (see details in in Materials and Methods).
We also inferred from your concern that the statistics should be more clearly presented. While some statistical analyses were previously included in the Supplement, we have now reorganized the figure and Supplementary panels to provide a clearer quantitative picture and added a new table reporting the number and percentage of regions analyzed. Our statistical approach is described in Materials and Methods.

4. Overall the analysis of the Cut&Tag data appears superficial. It would be useful to expand on this and compare the number of peaks that go up versus down, and the intensity of these peaks in box plots or similar. What is meant by 'other genomic regions'? was there a different threshold for this analysis? It seems that for both K9me3 and K27me3 the basal level across the genome is increased - outside peaks. Is this correct?

In the revised manuscript ,we now provide a more quantitative analysis for changes in H3K9me3/H3K27ac and H3K27me3 levels (new Fig. 2). This includes (i) the number of regions that show an increase versus a decrease, and (ii) comparisons of change magnitudes to better capture the distribution of responses across regions and conditions.

By “other genomic regions” we mean 500-bp regions in which no peaks were called in either wild type nor Cdk1-RNAi by the peak calling algorithm. Because these regions did not contain peaks, we did not apply an additional peak-calling threshold to this category. We now renamed this class in our Figures as ‘500 bp regions with no called peaks’, and include a supplementary schematic illustrating shared, unique, and no peak regions, so the categorization are transparent.

Regarding the reviewer’s interpretation: For H3K9me3, our data support increased signal also in different regions: we observe significant enrichment not only in shared peaks, but also in Cdk1-RNAi unique peaks and more importantly also in ‘no peak’ fragments. This pattern could be consistent with heterochromatin spreading, but we did not perform an analysis resolving location of these regions with respect to existing peak regions or with respect to heterochromatic domains. We thus mention it but refrain from discussing it in this context.

Yet, in contrast, for H3K27me3, significant enrichment is restricted to shared peaks and peaks unique to Cdk1-RNAi, with no enrichment in ‘no peak’ fragments, consistent with the more localized gene-silencing function of H3K27me3 that would not spread to adjacent domains.

The H3K9me3 snapshot show rather limited changes compared to the IF results and H3K27me3 - can the

authors comment? Would it be relevant to compare the behavior across repetitive regions versus repressed promoters?

We agree that the H3K9me3 CUT&Tag snapshot shows more modest changes. Please remember that fewer than ~30% of cells in this tissue express Cdk1-RNAi (due to GAL4 driver), so our CUT&Tag performed on the entire discs averages wild type and Cdk1-RNAi expressing cells, thereby diluting locus-specific changes and reducing sensitivity. The snapshot in the main figure highlights a euchromatic region, where H3K9me3 is generally low. However, we also detect changes at heterochromatic domains (see snap shot of Chr 2R below).
But yes, we completely agree that it would be interesting to compare H3K9me3 behavior at repetitive regions versus repressed promoters, but we have not performed this stratification here. We feel that such an analysis is better performed in more sensitive systems where 100% of cells express Cdk1-RNAi, and the range of detectable changes (especially at promoters) is larger.

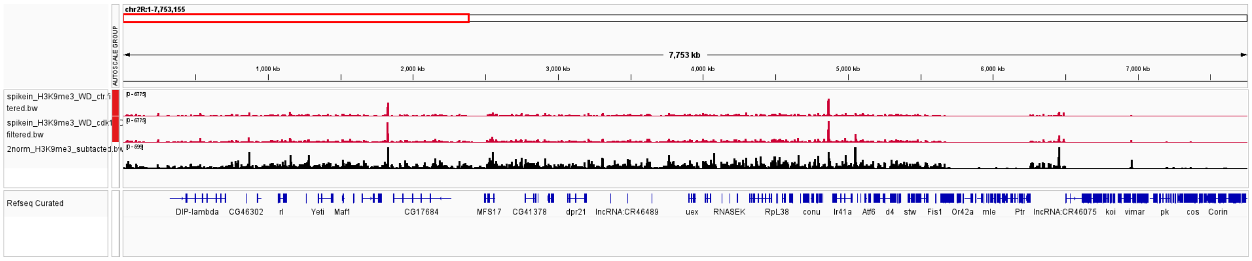

Please also include annotations on the axis of all plots with genomics data.

We reduced the number of profile views to one, and removed the gene track due to truly valid legibility concerns. Instead, we now highlight the location of the Hox-gene cluster and annotate the chromosome position in base pairs.

5. The paper would benefit substantially from being more quantitative. All key conclusions from IF data should be supported by quantification across replicates and statistics. This is relevant for several figures (Fig 3, Fig 4,..) .

We have now added quantification with statistical analysis across biological replicates for the IF experiments in Figures 1–5 (including Figs 3 and 4 highlighted by the reviewer). The corresponding figure legends have been expanded to describe the quantification and statistics. In the new source data file, we supply all relevant quantification data points, replicates and statistics. We hope these additions address the reviewer's concern.

Please also make sure all IF panels are fully annotated. It would also be helpful if relevant cells were marked by arrows or circles on more of the images.

We revised all panels to improve annotation (including genotype/condition and relevant markers), as well as labels and fonts for readability. Specifically, we have improved labeling across all microscopy figures by bolding the fonts and adding a black outline to enhance visibility against IF backgrounds. In addition, we added arrows to highlight the relevant cells in images, where this aids interpretation.

Please note that our layout choice intended to avoid overlabeling and preserve image clarity: for several panel blocks we place an IF label only on the first panel of the block and implied it to the corresponding grouped images. We have retained this format for image transparency but hope that each block is clearly delineated so that the shared labels are unambiguous. We hope these changes address the reviewer's concern.

6. The conclusions regarding gene expression are also based on IF patterns that are not quantified. This does not exclude changes in gene expression, especially more subtle and heterogeneous ones that would not lead to strong changes in protein levels. Quantitative data should be provided, preferentially on the mRNA level.

We completely agree with the reviewer that the expression patterns shown cannot exclude subtle changes, particularly at the mRNA level. Please note that our goal with this experiment was to assess the functional endpoint relevant for the tissue: expression of cell fate markers.
Moreover, these experiments were intended as a supporting observation, and based on our previous work, we did not expect changes in the expression of cell fate markers. The community has long examined the relationship between cell cycle and differentiation. In an otherwise normal imaginal disc, genetically extending or shortening the cell cycle has not been observed to alter developmental fate, at least by superficial analysis, for example (Mata et al., 2000, Neufeld et al., 1998, Weigmann et al., 1997, Gerlach et al., 2019). By contrast, fate changes have been reported when the cell cycle is manipulated in the context of tissue damage and compensatory proliferation, where the strong concurrent activation of damage-response pathways such as JNK and JAK/STAT (and Wnt) facilitates transdetermination (Worley et al., 2018, Repiso et al., 2013, Klebes et al., 2005, Lee et al., 2005, Sustar and Schubiger, 2005, McClure et al., 2008, Schubiger et al., 2010). Based on these studies, we expected that cell-cycle manipulation in the absence of strong tissue stress signals would be insufficient to change fates. We included this reasoning and citations now in the discussion to better support our conclusions.

7. The results on H3K27me3 are consistent with earlier findings that H3K27me3 accumulate in G1 /G0 arrested cultured cancer cells (Alabert et al., 2015) and a recent more elaborate study on H3K27me3 in ESCs (Trouth et al., 2025). Please include a discussion on this: the new work is in vivo and in a different organisms so discussing these findings together will help establish the general principles. H3K9me3 was not found to accumulate in G1/G0 arrested cells (Alabert et al., 2015), but rather in S phase arrested cells experiencing replication stress (Gaggioli et al., 2023). Please also discuss these differences. In relation to H3K9me3 regulation, the authors could also consider discussing DOI:10.1126/science.adq7408

We had already cited (Alabert et al., 2015, Trouth et al., 2025) in the first version of the manuscript, and now also include them in the revised discussion. Since we removed the idea of replication stress from the manuscript, and focus on S-phase regulation of H3K27ac, we do not discuss (Gaggioli et al., 2023) but included (Zhou et al., 2025) in our introduction.

8. In the abstract first line, do the authors mean epigenome integrity rather than genome integrity?

We initially used genome integrity as an umbrella term to describe both genetic and epigenetic stability. Because we have now removed the replication-stress context, we revised the first line of the Abstract to epigenome integrity.

9. The authors include a speculation on senescence in the results section as well as in the discussion. It should be removed from the results.

We have edited the Results to focus on data-driven statements and moved the senescence-related speculation to the Discussion.

Reviewer #2:

Nogay et al. show that levels of some histone modifications, but not others, are linked to the cell cycle in Drosophila wing imaginal discs. H3K9me3 and H3K27me3 accumulate as the cell cycle lengthens, and adopt to insulin signalling. By contrast, H3K27ac levels are highest in early S-phase and decline with longer cell cycles. Knock-down of the H3K27 acetylase CBP results in G2 arrest, and phospho-H2Avariant (gammaH2Av) accumulates in H3K27R mutant clones, indicative of replication stress upon H3K27ac reduction.

Overall, the paper presents a series of elegantly designed and well-explained experiments relying mainly on immunofluorescence in a Fly-FUCCI background to reveal a link between cell cycle and some histone modifications. The data are solid and will be of high interest to a wide community of biologists.

Comments

1. It is very intriguing that only some histone modifications are sensitive to perturbation of the cell cycle. In particular, H3K27ac is sensitive whereas H3K18ac and H4K8ac that are acetylated by the same enzyme (CBP/nejire) are not. Can the authors speculate on the reasons for this?

Although all three marks can be acetylated by CBP/Nejire, H3K27ac is most prominently associated with active enhancers and promoters, whereas H3K18ac, and H4K8ac are often described as more general euchromatic acetylation marks, suggesting that they maybe subject to additional layers of regulation do not equivalently control all three marks. In addition, cell-cycle or co-factor dependent modulation of acetylation, as well as deacetylation by HDACs may affect these three marks with different sensitivity. Supporting this idea is our finding that expression of Nej RNAi completely abolished H3K27ac within one cell cycle, whereas residual H3K18ac, and H4K8ac can still be detected (see Fig S5.2). Thus, we do not exclude the possibility that H3K27ac is regulated by additional factors whose activity or recruitment varies across the cell cycle. We now emphasize this point in the discussion.

2. Expression of tested Polycomb-targets are not affected in Cdk1i or by Dp, E2F1 overexpression. Is this related to a block in cell cycle progression or that levels of H3K27me3 do not change enough? I would like to see a quantification of the H3K27me3 signal in the E(z)i experiment to be able to compare it with the Dp, E2F1 over-expression. Is the reduction in H3K27me3 achieved after 24h E(z)i sufficient to cause Polycomb-target de-repression?

We addressed this directly by comparing H3K27me3 reduction achieved by E(z)-RNAi versus Dp, E2F1 overexpression within the same 24 h window, assessing AbdB staining as a PcG target gene read-out. In both conditions, H3K27me3 is reduced but not fully lost, and the extent of H3K27me3 reduction is comparable between E(z)-RNAi and Dp, E2F1 overexpression. Nevertheless, neither condition is sufficient to derepress PcG target gene AbdB, indicating that the partial loss of H3K27me3 reached after 24 h is insufficient to mediate Polycomb target gene activation.
We would be happy to include this result in the manuscript, if you deem this necessary. We were trying to keep the main story line focused and were unsure if this result would distract.

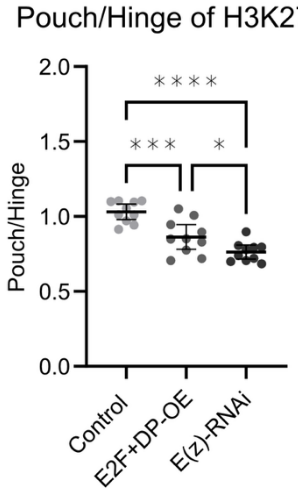

Quantification of relative signal intensities for H3K27me3 presented as pouch-to-hinge ratios (rn^{ts}-to-wild-type-cell ratio) in Dp, E2F1-coexpressing and E(z)-RNAi-expressing discs. Mean and 95% CI is shown. Statistical significance was tested using Ordinary one-way ANOVA followed by Tukey's post-hoc test for multiple comparison (control discs: n=10, Dp, E2F1-coexpressing discs: n=10, E(z)-RNAi-expressing discs: n=10). Adjusted p=0.0005 for Control vs E2F+DP-OE; p<0.0001 for Control vs E(z)-RNAi and p=0.0374 for E2F+DP-OE vs E(z)-RNAi.

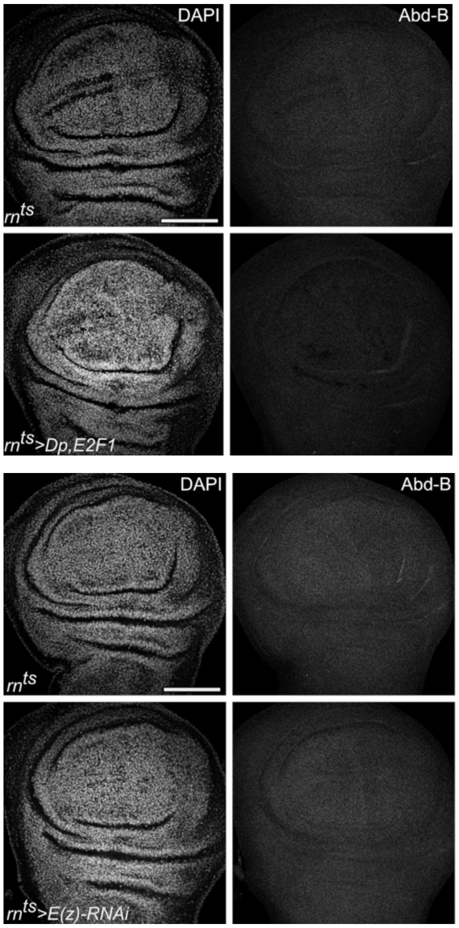

3. On a similar note, the Cavalli lab showed that JAK-STAT signalling and its target ZFH1 become irreversibly up-regulated upon transient knock-down of the PRC1 component Ph (PMID: 38658752). Is this pathway affected upon cell-cycle perturbation?

In our experience, cell-cycle perturbations elicit a mild but detectable tissue stress response, which includes JNK activation (please see figures supplied in response to Reviewer 3 comment 2). JNK activation could induce JAK/STAT signalling via Upd upregulation - independent of direct Polycomb impairment and H3K27me3 downregulation. With our current set up we would not be able to distinguish between these two possibilities. Because we have not directly assayed JAK/STAT pathway activity in this study, we would like to refrain from introducing any speculation about this pathway in the discussion of this manuscript.

4. If insulin signalling affects the abundance of metabolites such as SAM, why is not H3K4me3 levels altered? Metabolism should also affect the amount of acetyl-CoA, so how does H3K27ac look like?

We think Insulin signaling can, in principle, impact all of these marks via metabolite availability. Yet, our data suggest that in fast-cycling InR^{CA}-only expressing cells, H3 methylation levels do not change, consistent with a tight balance between metabolism-set modification levels and dilution or turnover through division. By contrast, in cell-cycle arrested InR^{CA}-expressing cells, H3K9me3 and H3K27me3 become more elevated than in Cdk1 RNAi alone. H3K4me3 may be comparatively buffered because it is concentrated at active genes and is closely coupled to ongoing transcriptional activity; therefore both its deposition and removal might be very dynamic and controlled, which could compensate for moderate metabolite changes (see also (Zheng et al., 2014)). In contrast, repressive chromatin associated with H3K27me3/H3K9me3 may be less tightly supervised in arrested cells, where maintaining a default or active silent state may require fewer dynamic regulatory inputs than sustaining active transcription.

Regarding acetylation, we agree that insulin-dependent metabolism could also affect acetyl-CoA. We tested H3K27ac (see figure to the right) and, again, do not detect strong changes in fast cycling InR^{CA}-only cells, again suggesting a balance between metabolite-driven input, acetylation, and division. However, Cdk1-RNAi reduces H3K27ac both with and without InR^{CA}. Thus InR^{CA} and a potential higher acetyl- CoA supply does not restore H3K27ac levels. One explanation is that the H3K27ac decrease under Cdk1-RNAi is not primarily limited by acetyl-CoA availability, but instead reflects the cell-cycle-dependent regulation of H3K27ac writers/erasers or their recruitment (see Fig 5). Again, we did not include it in the manuscript to maintain a tight focus in Fig 4 on methylation and in Fig 5 on acetylation.

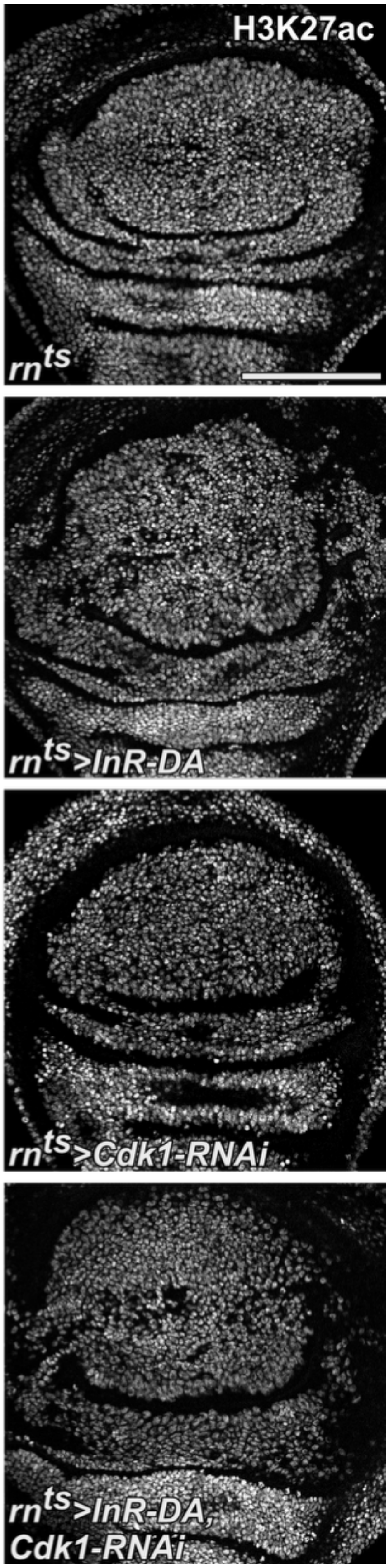

5. Levels of total H3 and all acetylations are elevated in early S phase (Fig. S5.1 and 2). How come then that only H3K27ac is increased in cells with fast cell cycles?

Please allow us to clarify: we find that total H3 is highest in G1 and early S-phase, whereas total acetylation peaks in early S phase. H3K27ac also rises in early S (consistent with the overall acetylation), yet it remains elevated in late S compared to total acetylation, which declines. We now moved the quantification of acetylation dynamics into the main Fig. 5 to emphasize this difference.

The distinction between early and late S is important: many E2F/DP-expressing cells are in late S phase, where total acetylation is low but H3K27ac remains high. Thus, a bulk increase in H3K27ac can arise from a shift in cell-cycle phase distribution to a relatively higher late-

S phase percentage. We also note that there was a mild trend for total acetylation elevation in E2F,DP, it just was not significant.

6. What is known about how HDAC1 is regulated during the cell cycle? The previous literature on the effects of HDAC1 knockdown on the cell cycle should be discussed.

To our knowledge, relatively few studies have directly examined cell-cycle–dependent regulation of HDAC1 itself (e.g., systematic changes in abundance, localization, or post-translational modifications across phases). We have added a brief discussion of evidence for mitosis-linked regulation (including phosphorylation by mitotic kinases such as Aurora kinases and CK2) and for S-phase-associated roles in replication-coupled chromatin regulation. Most literature addresses the reverse relationship about how HDAC1/2 depletion or inhibition affects cell-cycle progression via histone and non-histone substrates linked to replication stress or mitotic phenotypes (for example (Munoz et al., 2024, Parveen et al., 2023, Segre et al., 2016, Loponte et al., 2016, Kehrli et al., 2016, Jamaladdin et al., 2014, Khan et al., 2013, He et al., 2013, Bhaskara et al., 2013, Marks, 2010, Li et al., 2006, Pile et al., 2002)). We know state in the manuscript that: '...While CBP/nej has been reported to be regulated by cyclin-dependent kinases at the G1/S transition (Ait-Si-Ali et al., 1998), HDAC1 is best known to undergo mitosis-associated phosphorylation regulating chromatin association and enzymatic activity during mitotic entry (Loponte et al., 2016, Segre et al., 2016, Khan et al., 2013, He et al., 2013).'

7. The EdU signal (Fig. S4.1E,D) in InR-DA and gammaH2Av levels in H3K27R mutant clones (Fig. 6K) should be quantified.

After discussions with the editor, we have removed Figure 6 from the paper.

Minor comments

1. Line 162: there are two Buttitta et al references from the same year.

Corrected.

2. In Fig. S2.1, the counts for the unique peaks are very similar to "other genomic regions", questioning if they are really new peaks or just background signal.

We revised our presentation of how we define and use the term "other genomic regions". In our analysis, "other genomic regions" refers to genomic intervals that were not called as peaks by the peak-calling procedure, but for which we nonetheless quantified changes in levels of modifications. Please refer to new Fig 2 and new Fig S2 as well as updated result and methods sections with (hopefully) improved presentations of this category.

3. The nuclei in inflammatory senescent cells look different from peripheral quiescent cells (Fig. S4.2). Can the authors be sure that higher H3K9me3 signal is not simply due to this difference rather than higher total levels of H3K9me3 in these cells?

We agree that nuclear morphology differs between inflammatory senescent-like cells and peripheral proliferating or quiescent cells, for reasons that we do not understand yet. The nuclei in senescent-like are typically enlarged and the DAPI-stained chromatin looks different, a phenotype also often observed in

neoplastic tumor models (including but not exclusive to PRC1 mutations). Such difference could, in principle, influence the apparent distribution of H3K9me3. However, H3K9me3 within the senescent nuclei is not only more widespread but also visibly higher in intensity. We therefore interpret the data as consistent with increased H3K9me3 levels in these cells, while acknowledging that we cannot formally exclude contributions from ultrastructural reorganization that affects apparent signal. Of note, previous publication also report quantifiable changes to H3K9me3 in senescent cells in imaginal discs (Zang et al., 2025, Ito and Igaki, 2021), suggesting that this is a common feature of senescent cells in many model systems, even in Drosophila.

4. Does nejire OE cause an increase in wing disc size or is the size difference in Fig. S5.1G-J due to rearing conditions?

We did not detect an increase in wing disc size upon nejire OE in our experiments: across staged and sibling-matched cohorts reared under identical conditions, disc size remained within the normal range of developmental variability, which Fig. S5.1G–J reflects . In response to this comment, we have replaced the panel with another representative image with comparable disc size between conditions.

5. Is cell cycle phase distribution affected by nejire OE?

We did observe that nejire overexpression alters the cell-cycle phase distribution. However, unlike the phase-dependent changes in H3K27ac levels, this shift in cell cycle dynamics is not phenocopied by HDAC1-RNAi (see quantifications of n=6-10 discs in each genotype below), suggesting that the cell-cycle effects of nejire overexpression are not straightforwardly attributable to the same mechanism. Given that manipulating nejire and HDAC1 can have pleiotropic consequences on the cell cycle, we have chosen not to include these data in the revised manuscript to avoid speculation and overinterpretation.

Cell cycle - nejOE

	Control Ratios	nejire-OE Ratios
G1	0.175	0.252
Early S	0.117	0.215
Late S	0.137	0.188
G2	0.571	0.345

Cell cycle - HDAC1-RNAi

	Control Ratios	HDAC1-RNAi Ratios
G1	0.165	0.114
Early S	0.079	0.039
Late S	0.141	0.091
G2	0.615	0.757

Cell cycle – HDAC1-OE

	Control Ratios	HDAC1-OE Ratios
G1	0.161	0.136
Early S	0.074	0.078
Late S	0.124	0.131
G2	0.640	0.655

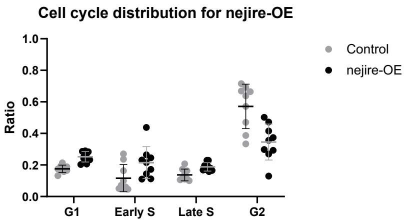

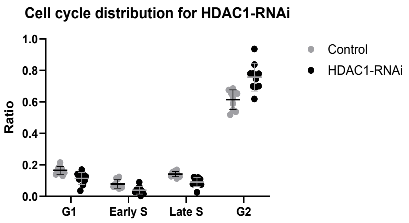

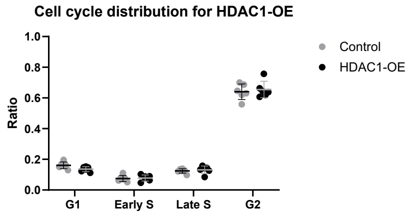

6. Please comment on the difference between Fig. 5J and 5J', is there a bigger effect at other cell cycle stages?

We now revised Fig 5 and included all cell cycle stages. Please see our response to reviewer 1 comment 2 for a brief summary, as well as the updated Fig 5 and the new corresponding result and discussion section.

7. What is the evidence for the statement on lines 372-373: "which is consistent with the reduced CBP/nej substrate specificity for these two residues". Please give references.

We agree that the original wording was misleading and implied prior biochemical evidence for reduced CBP/Nej substrate specificity. Our statement was instead based on our data (Fig. S5.2G–N), where after nejire knockdown H3K27ac is completely lost within one cell cycle, whereas H3K18ac and H4K8ac levels are only partially reduced. During revisions, this section has now been removed.

8. Can the signal in Fig.6F,G also be normalized on the level in the hinge region? The difference between hinge and pouch is hard to appreciate.

After discussions with the editor, we have removed Figure 6 from the paper.

Reviewer #3:

PLoS Biology Review: In this manuscript, the authors utilize fly genetics and the developing wing imaginal disc to characterize cell cycle associated changes in a set of histone post-translational modifications (hPTMs), suggesting roles for cell cycle associated chromatin dynamics in metabolism, senescence, and genome stability. The manuscript is well written and with interesting hypothesis, however, in its current state the analyses presented do not adequately and robustly support the author's conclusions. In many cases, proper quantification with statistics are missing.

Major Critiques:

* The authors provide a sophisticated repertoire of quantification methodologies that, when used, support their images and writing effectively. Unfortunately, these methodologies are used inconsistently and sparingly throughout this manuscript, with many conclusions being made qualitatively from individual images. For example, the primary GAL4 driver used in this manuscript, rn-GAL4, is expressed exclusively in the wing pouch, data from which can be normalized to the wing hinge, which acts as an internal control that the authors utilized in Figures 1U-W. This type of quantification is largely absent from the rest of the manuscript. If present, these qualifications would provide insight into the visually subtle changes in hPTMs described by the authors and would likely provide support for their conclusions.

We have now provided new quantifications for all figures of the paper.

* Given the spatial specificity of rn-Gal4, would be better to generate real mosaic clones using ubiquitous Gal4 drivers to compare RNAi or overexpression clones with adjacent controls. This strategy is particular powerful when using immunostaining signals to quantify and draw conclusions.

We agree that mosaic analysis can be a powerful approach for immunostaining-based quantification because it provides an internal adjacent control. We initially performed flip-out mosaic experiments for this reason and observed the same changes in histone modification signals in cell-cycle-manipulated clones we tested compared to their neighboring wild type cells. We include a representative mosaic-clone data set for the reviewer (UAS-pnt), demonstrating the same changes in H3K27 and H3K9 modifications as observed with rn-Gal4 (<u>see 1st figure below</u>).However, we ultimately adopted the rn-Gal4 tissue driver because it provides more reproducible spatial and temporal induction of genotypes across discs. With the flip-out system, clone position and size vary substantially between samples, and several of the cell-cycle perturbations we use strongly influence clone morphology, clone growth and include phenotypes consistent with clone growth disadvantages, complicating the interpretation and quantification (<u>see 2nd figure below</u>). These problems were strongly reduced when using a broad tissue driver.

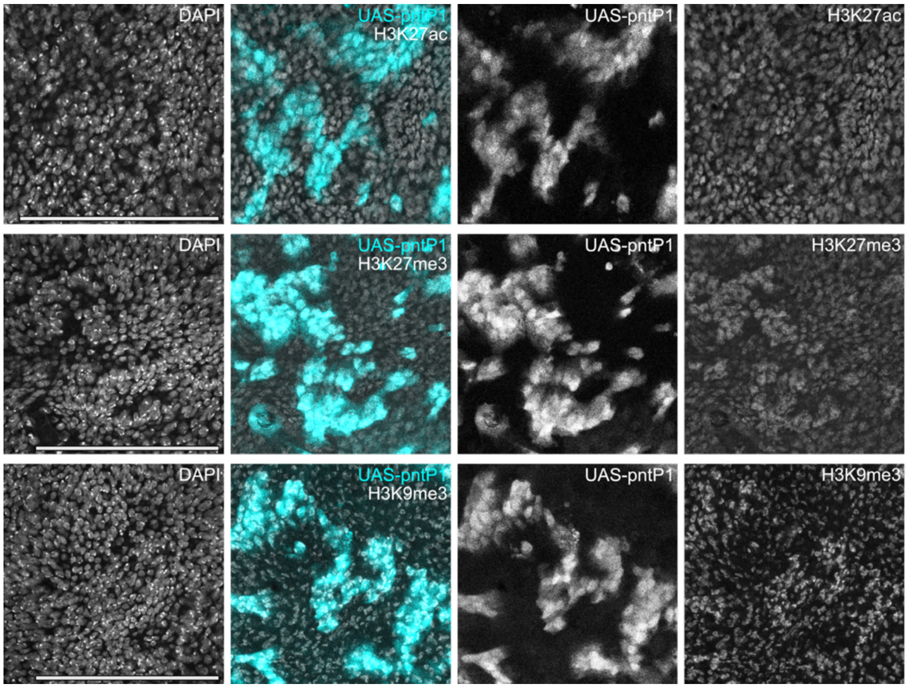
DAPI
UAS-pntP1
H3K27ac
UAS-pntP1
H3K27ac
DAPI
UAS-pntP1
H3K27me3
UAS-pntP1
H3K27me3
DAPI
UAS-pntP1
H3K9me3
UAS-pntP1
H3K9me3

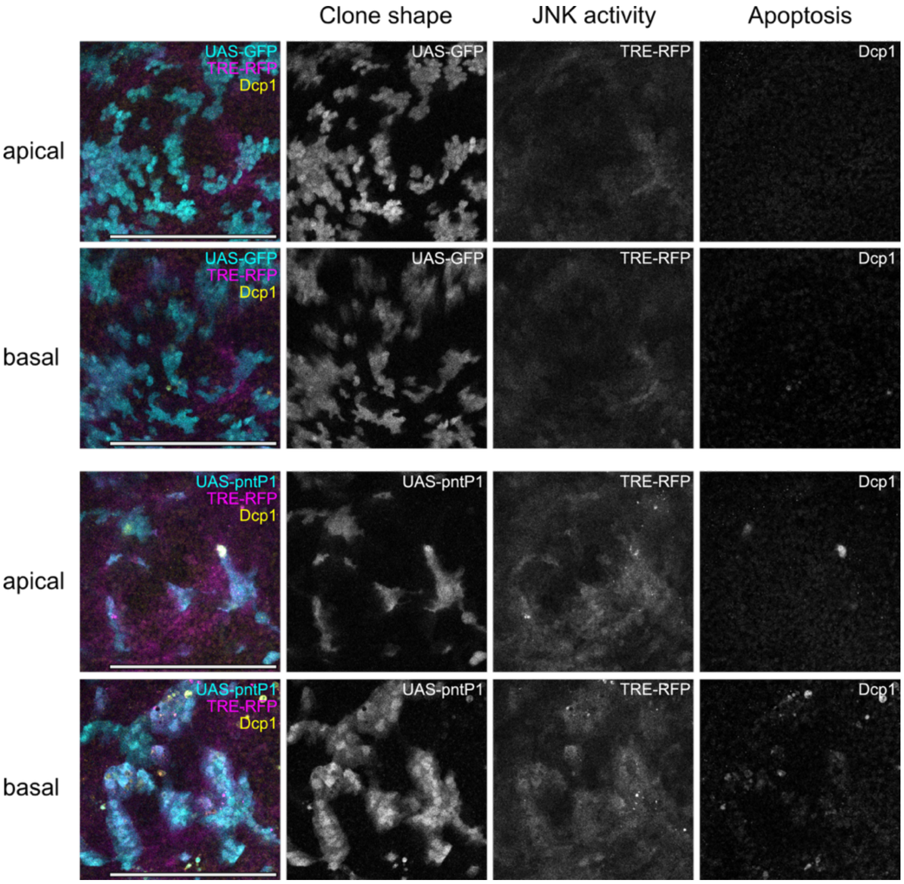
Clone shape
JNK activity
Apoptosis
apical
basal
apical
basal
UAS-GFP
TRE-RFP
Dcp1
UAS-pntP1
TRE-RFP
Dcp1

* Another example is the normalization of hPTMs, which in this work are mainly on H3. Therefore, normalization of each hPTMS by H3 signals would be more informative. In fact, the H3 by itself should also reflect cell cycle dynamics, as noted by authors in 351-353: "Of note, fluctuation of H3 and total lysine acetylation in our microscopy assays can be mostly explained by S-phase dependent histone synthesis and histone acetylation, as well as fluctuating morphology of interphase nuclei".

We extensively assessed if and how to best normalize hPTMs but found that each approach was limited for different reasons. For example, total H3 signal is highest in G1 and early S-phase and likely reflects histone production in preparation for DNA replication. Yet, the increase of H3 levels in G1 probably does not reflect chromatin-incorporated H3 carrying chromatin-associated PTMs. In addition, we found that our cell-cycle manipulations have independent effects on total H3 levels in our immunofluorescence assays (IF). For example, H3 is elevated in cdk1-RNAi cells but reduced in UAS-pnt cells, and stays fairly normal in UAS-Dp,E2F cells (see figure below). If used as a normalizer, the high H3 signal in cdk1-RNAi would account for increased methylation but would exaggerate the apparent decrease in acetylation, whereas the low H3 signal in UAS-pnt would have the opposite effect. Importantly, our CUT&Tag data suggest that the elevated H3 signal in IF may not equate the H3 chromatin-incorporated pool: the H3-normalized CUT&Tag profiles for hPTMs in cdk1-RNAi recapitulate the IF-detected increase in H3K9/H3K27 methylation and decrease in H3K27ac, which would be difficult to reconcile with a simple global increase in nucleosome-incorporated H3.

In summary, we observe a consistent PTM signature in IF for widely different cell cycle accelerating or cell cycle stalling genotypes (see Fig 1 and Supplements) - supporting their strong link between H3K9 and H3K27 modifications . In addition, our Cut&Tag data reveals that total H3 levels in IF is not a reliable proxy for chromatin-incorporated H3. For these reasons, we do not normalize to total H3 IF signals, as total H3 IF levels would not scale with levels of chromatin-incorporated H3.

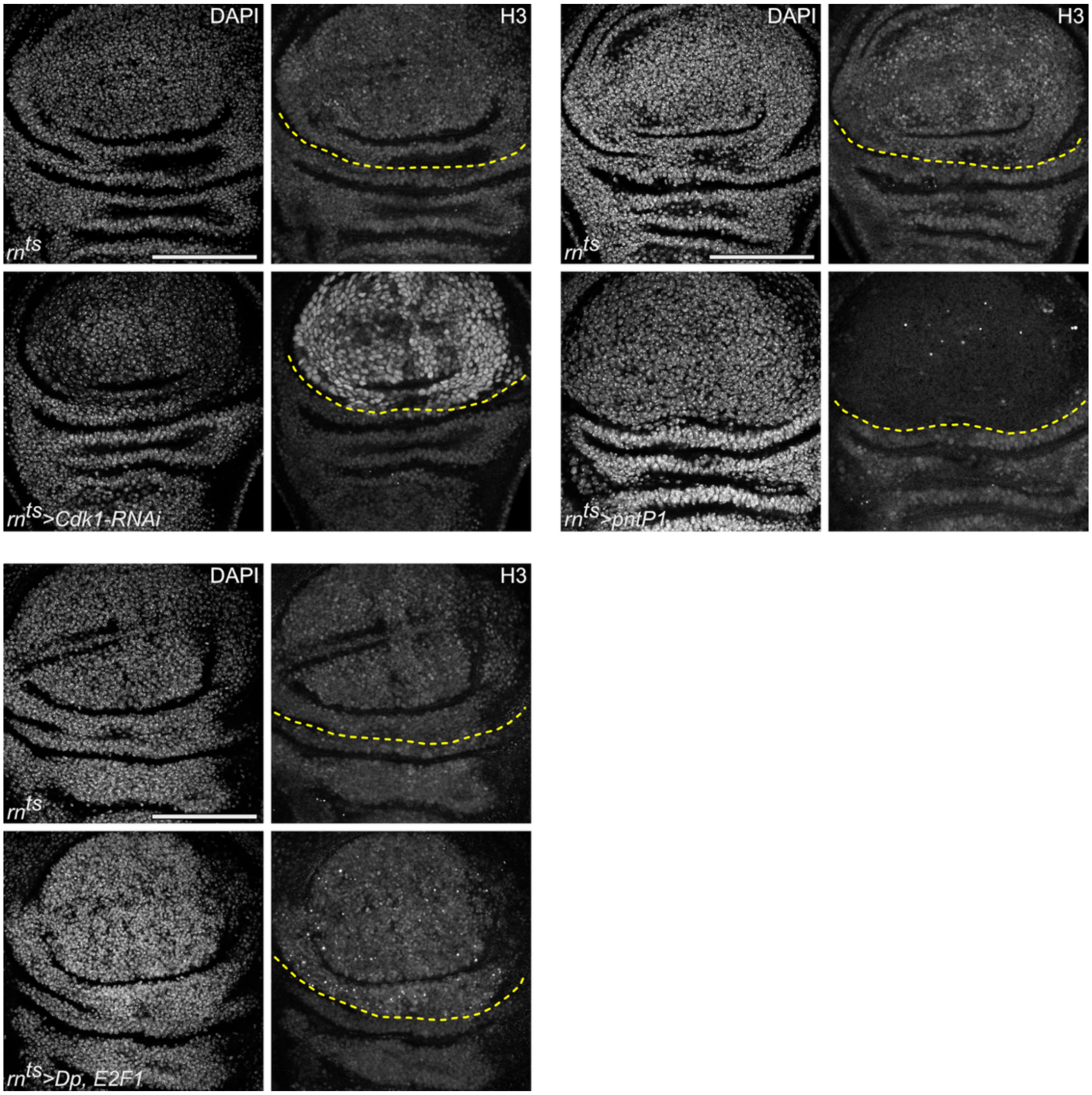

* Similarly, the authors use FUCCI to distinguish cell cycle phases in their analysis of hPTMs. The authors use a thresholding-based method for classifying cell cycle phase, but are inconsistent in providing quantification, often providing only large, heterogenous fields of view like seen in Figures 3A-C. This quantification is also necessary for all FUCCI data.

We completely agree that quantitative classification is important when FUCCI is used to assign cell-cycle phase at the single-cell level. However, in several key experiments (especially Fig. 3 and Fig. 4), our primary goal was to document well-established cell-cycle domains in the wing disc (e.g., dorsal-ventral boundary, hinge-associated arrested regions, or the rn-GAL4-manipulated domain). These domains converge on reproducible (and often homogeneous) cell-cycle state. For Fig. 3 in particular, the regional cell-cycle patterns have been extensively characterized in prior work by multiple groups, which we cite. For Fig. 4, we distinguish between a normal, accelerated, and arrested cell cycle, but we do not make claims about quantitative differences between individual cell cycle phases. Thus, for many experiments, presenting large

fields of view is intentional: it preserves tissue context and makes the correspondence between spatial domains of expected cell-cycle behavior and the changes in hPTM levels immediately comparable.

However, to address your concern directly, we now include visual guides in Fig. 3 to direct the reader to the key cell-cycle-arrested domains. In addition, we have expanded quantitative analyses throughout the revised manuscript: hPTMs are now quantified in Figs. 3 and 4 (and their Supplements), and FUCCI-based cell-cycle distributions are quantified in Fig. 1 and 5.

* Distinction between early and late S-phase appears to be arbitrary based on the author's thresholding approach. As late S-phase is associated with replication of constitutive heterochromatin, validation that these thresholds can distinguish between early and late S-phase can be achieved via EdU incorporation with anti-H3K9me3 antibody staining, or other image-based methods to distinguish early vs. late S-phase nuclei.

We apologize if our description gave the impression that the early and late S-phase classification was arbitrary. Our thresholds are based on prior validation of the FUCCI reporter in our lab, where we correlated FUCCI states with EdU incorporation patterns that distinguish euchromatic (early S-phase) from heterochromatic (late S-phase) DNA replication (see Supplements in (Crucianelli et al., 2022, Cosolo et al., 2019). In these datasets, FUCCI-negative cells show euchromatic EdU patterns (thus classified as early S-phase), whereas FUCCI-red show EdU enrichment in heterochromatic domains (consistent with late S-phase replication). Thus, our early/late S-phase assignment is anchored to EdU-based replication-pattern validation rather than being defined solely by an arbitrary intensity cutoff. To reflect this in the manuscript, we have now added representative FUCCI-EdU co-stained image to the Supplements (Fig S5.1 A,B) and updated the Methods to describe the rationale for the thresholding approach.

Other Critiques:
* Labels on microscopy images are very hard to read and sometimes missing.

We agree that some labels were difficult to read. We have improved labeling across all microscopy figures by bolding the fonts and adding a black outline to enhance visibility against IF backgrounds. The impression of missing labels likely arises from our layout choice intended to avoid overlabeling and preserve image clarity: for several panel blocks we place a label only on the first panel of the block and implied it to the corresponding grouped images. We have retained this format for image transparency but hope that each block is clearly delineated so that the shared labels are unambiguous.

* Fig 2A-B: When comparing the WT and Cdk1-RNAi histograms the change in peak height is not always obvious, which makes the "change" histograms look exaggerated. What may help is having a dotted line on the histograms at the WT peak maxima, which will act as a ruler in each histogram for differences in peak height. Also, where are the statistics of these comparisons?

We agree that peak-height differences are subtle to judge visually, as only 30% of cells express Cdk1-RNAi due to spatially restricted rn-GAL4 activity. Thus, the signal reflects an average of RNAi-expressing and wild-type cells and is expected to dilute locus-specific effects. We appreciate the suggestion of adding a dotted "ruler" line at the WT maximum. We tested this, but it reduced readability in our figure layout. Instead, we think that the "change PTM" tracks convey the differences more clearly. We also agree that the statistics should be more clearly presented. While some statistical analyses were previously included in the Supplement, we have now reorganized the figure and Supplementary panels to provide a clearer quantitative

picture and added a new table reporting the number and percentage of regions analyzed. We moved the genomic plots to the bottom of the image as a representative example. Our statistical approaches are described in Materials and Methods.

* Fig 3: The locations of A-C and D-F on the wing disc are unclear, especially to a non-drosophilist. A diagram of the wing disc with these regions highlighted would be helpful for the reader. Again, where are the quantification and statistics of these data?

We have added clearer visual guides in the figure (including colored arrowheads) to indicate the corresponding domains on the wing disc. In addition, we now include quantification and statistical analyses for these datasets in Fig. 3 and the associated Supplement.

* Fig S4D-E: The authors state that there is "high EdU incorporation" in the InR-DA expressing wing pouch, however, visually the EdU in this tissue looks identical if not in fewer cells compared to the control. The figure legend states that this is based on high EdU intensity, however, this could result from different Click-iT reaction efficiencies. More appropriate analyses would be a S-phase index and/or colocalization analyses with between EdU and DAPI. Regardless, quantification is needed.

Of course. We previously quantified this phenotype, which we now also cite in the text (Fig. S8 in Crucianelli et al., 2022). We copy the data here in our responses and hope that this addresses your concern.

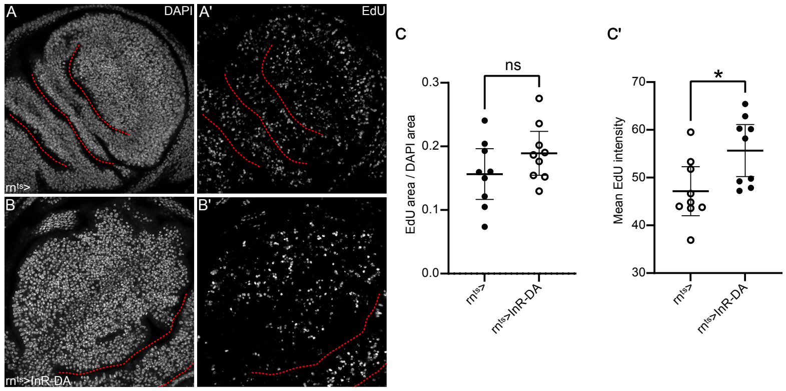

* Fig 4E: The authors state that there is no change in H3K27me3 compared to the control (4D), but a region of the wing pouch next to the dotted line looks like it has lower H3K27me3 than the control. Quantification would provide clarity on this.

We now provide quantifications in Figure 4.

* Fig 4M: The authors state that H3K4me3 does not change between 4L-O, however, it looks lower in 4M. Quantification would clarify this.

We now provide quantifications in Figure 4.

* Fig S4.2H: It is unclear if there is overlap between the inflamed region and the cyan "proliferating" region. SA-β-Gal or TRE-RFP included in these images would clear this up.

We have now added an overlay including TRE-RFP in Fig. S4.2 to visualize high TRE-RFP–positive (senescent-like/inflamed) cells, peripheral low TRE-RFP cells (strongly overlapping with the proliferative domain), and TRE-RFP-negative quiescent cells, thereby clarifying the spatial relationships between these regions.

* Figure 6F-G: The authors report that upon nej knock-down there is an increase in γH2Av signal within the wing pouch, lending support to the hypothesis that nej protects the genome from replication stress induced DNA damage. Though γH2Av signal does appear more uniform in the nej RNAi wing pouch, it is unclear as to whether the signal within wing pouch nuclei is in fact greater than the control or even the wing hing of the same wing disc. Quantification is necessary to support this conclusion.

After discussions with the editor, we have removed Figure 6 from the paper.

Reviewer #4:

The manuscript entitled "Cell cycle dynamics regulate H3K27and H3K9 modifications" uses the wing imaginal disc to assess the overall connection between cell cycle progression and changes to the chromatin landscape. Using a range of physiological and altered conditions, the authors note associations between cell cycle phases and overall levels of H3K9me3, H3K27me3, and H3K27ac. At least one modification, H3K4me3, remained unchanged. These changes occur at distinct times during the cell cycle and appear to be regulated separately. In addition to carrying out staining to examine the "big picture" changes to chromatin that occur, the distribution of H3K27me3 and H3K9me3 were examined in more detail using CUT&TAG. Overall, the data described in this manuscript provide in vivo insights into the dynamic and complex relationship that must exist to allow for proliferation and genomic fidelity.

Several parts of the manuscript could be clearer or expanded upon to strengthen the story.

1. In Figure 2, CUT&Tag data are shown comparing control and Cdk1 knockdown wing disc cells. In the genome tracks shown in A, the "change" tracks are somewhat deceptive, as they are not displayed at the same scale as the control and knockdown tracks above them. This makes it seem that the log2 fold changes are more dramatic than they are. As an alternative, the tracks could be overlaid to show the difference. It was also unclear whether analyses were conducted to determine which changes across the genome were significantly different or if the analyses were purely qualitative.

We also realized that the statistics should be more clearly presented. While some statistical analyses were previously included in the Supplement, we have now reorganized the figure and Supplementary panels to provide a clearer quantitative picture and added a new table reporting the number and percentage of regions analyzed. Our statistical approaches are described in Materials and Methods.
We agree that our scaling of the “change” tracks overemphasize differences. We really did not want to mislead the reader: in the manuscript we indicated the differing scales explicitly to make the presentation transparent. The observed changes are relatively small in this dataset because our CUT&Tag was performed on whole wing discs in which only a subset of cells (≤ ~30%, due to spatially restricted rn-GAL4 activity) express Cdk1-RNAi. Thus, the signal reflects an average of RNAi-expressing and wild-type cells and is expected to dilute locus-specific effects. We include a scale-adjusted version of the plot in our response and are happy to discuss whether this should replace the current version. However, due to the reason discussed above we would prefer to visualize the changes as before and rely on the quantitative data now presented in Fig 2 to demonstrate robustness of the data.

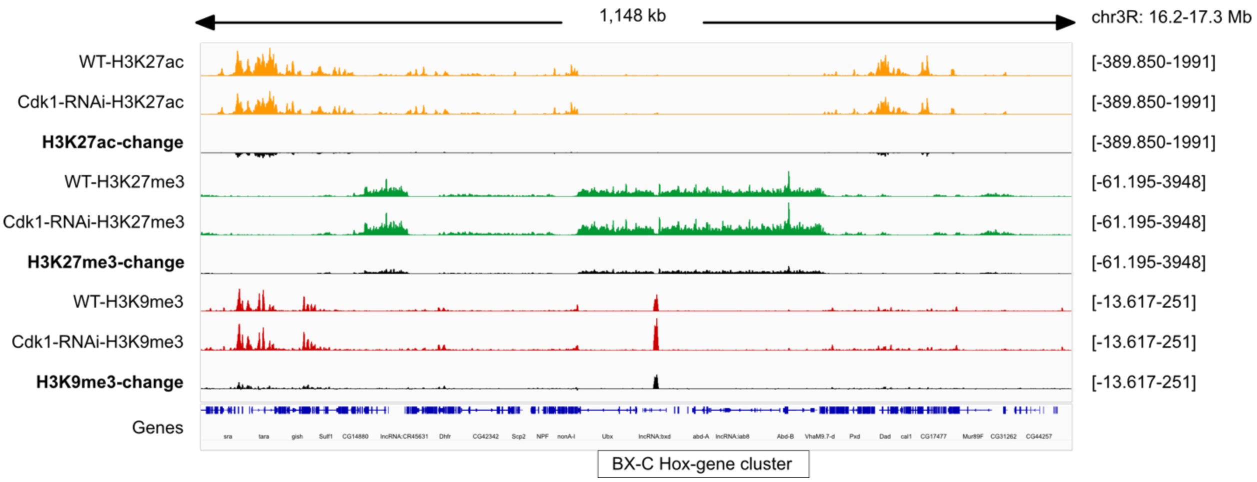

2. More information about the H3K27ac/H3K27me/K9me peak distribution would have been valuable. Is there any relationship with the greatest changes and regions of chromatin accessibility? Are ATAC-seq data available from wildtype animals that can be used for this? The authors also mention ORC complex in the discussion. Are there any binding datasets that could be interrogated?

We completely agree that it would be interesting to provide more information on the distribution of H3K27me3/H3K27ac/H3K9me3 peaks across genomic features (genes, promoters, repetitive regions, etc.). We did not perform this stratification here. We feel that this type of analysis would be much more informative in a more sensitive setting where ~100% of cells express Cdk1-RNAi, increasing the dynamic range of detectable changes. Given these sensitivity considerations and the scope of our study as a tissue-level analysis, we focused on a more general conclusions; especially since we do not detect cell fate changes associated with changes in gene expression programs that would beg us to look at specific gene-associated features (see Fig S3.2). However, we have made our datasets available for readers who wish to explore these molecular features in greater depth.

For the same reasons, we decided to forego ATAC-seq or additional analyses of chromatin accessibility, since we would remain limited by sensitivity. We explored to express cdk1-RNAi using more ubiquitous drivers, but these caused widespread lethality during the induction window, which introduces an additional layer of complexity to interpretation. We therefore settled on rn-GAL4, which we characterized throughout the paper by immunofluorescence, while acknowledging that this choice represents a compromise in terms of sensitivity for Cut&Tag analysis.

With respect to ORC, binding datasets are available (e.g., from modENCODE) but, to our knowledge, only from cultured Drosophila cell lines (Kc, S2, BG3), and not from in vivo settings and not for imaginal discs. To avoid presenting a speculative analysis based on correlations across different tissues and developmental stages, we decided not to pursue this further.

3. In Figure 4Q, a heatmap model is presented. Precisely what this means is unclear, particularly as not all methyl marks show changes.

We agree that the original presentation in Fig. 4Q was oversimplified, and we did not clearly specify that the model refers specifically to H3K9me3 and H3K27me3 (rather than all methyl marks). We have now corrected this by explicitly indicating H3K9 and H3K27 trimethylation in the figure and legend.
Fig. 4Q is intended as a hypothetical model to illustrate how histone trimethylation could be influenced by the independent and linked effects of manipulating the cell cycle and growth-promoting metabolism. The revised legend now reads: "Hypothetical model depicting the relationship between histone H3K9 and H3K27 trimethylation levels, metabolic rate, and cell division rate. When metabolic activity and cell division are proportionally coupled (black diagonal), histone methylation levels are maintained at stable levels. However, when the cell cycle is slowed or when metabolism outpaces cell division, methylation accumulates."

References

- AIT-SI-ALI, S., RAMIREZ, S., BARRE, F. X., DKHISSI, F., MAGNAGHI-JAULIN, L., GIRAULT, J. A., ROBIN, P., KNIBIEHLER, M., PRITCHARD, L. L., DUCOMMUN, B., TROUCHE, D. & HAREL-BELLAN, A. 1998. Histone acetyltransferase activity of CBP is controlled by cycle-dependent kinases and oncoprotein E1A. *Nature,* 396**,** 184-6.
- ALABERT, C., BARTH, T. K., REVERON-GOMEZ, N., SIDOLI, S., SCHMIDT, A., JENSEN, O. N., IMHOF, A. & GROTH, A. 2015. Two distinct modes for propagation of histone PTMs across the cell cycle. *Genes Dev,* 29**,** 585-90.
- BETTENCOURT-DIAS, M., GIET, R., SINKA, R., MAZUMDAR, A., LOCK, W. G., BALLOUX, F., ZAFIROPOULOS, P. J., YAMAGUCHI, S., WINTER, S., CARTHEW, R. W., COOPER, M., JONES, D., FRENZ, L. & GLOVER, D. M. 2004. Genome-wide survey of protein kinases required for cell cycle progression. *Nature,* 432**,** 980-7.
- BHASKARA, S., JACQUES, V., RUSCHE, J. R., OLSON, E. N., CAIRNS, B. R. & CHANDRASEKHARAN, M. B. 2013. Histone deacetylases 1 and 2 maintain S-phase chromatin and DNA replication fork progression. *Epigenetics Chromatin,* 6**,** 27.
- BUTTITTA, L. A., KATZAROFF, A. J., PEREZ, C. L., DE LA CRUZ, A. & EDGAR, B. A. 2007. A double-assurance mechanism controls cell cycle exit upon terminal differentiation in Drosophila. *Dev Cell,* 12**,** 631-43.
- COSOLO, A., JAISWAL, J., CSORDAS, G., GRASS, I., UHLIROVA, M. & CLASSEN, A. K. 2019. JNK-dependent cell cycle stalling in G2 promotes survival and senescence-like phenotypes in tissue stress. *Elife,* 8.
- CRUCIANELLI, C., JAISWAL, J., VIJAYAKUMAR MAYA, A., NOGAY, L., COSOLO, A., GRASS, I. & CLASSEN, A. K. 2022. Distinct signaling signatures drive compensatory proliferation via S-phase acceleration. *PLoS Genet,* 18**,** e1010516.
- GAGGIOLI, V., LO, C. S. Y., REVERON-GOMEZ, N., JASENCAKOVA, Z., DOMENECH, H., NGUYEN, H., SIDOLI, S., TVARDOVSKIY, A., URUCI, S., SLOTMAN, J. A., CHAI, Y., GONCALVES, J., MANOLIKA, E. M., JENSEN, O. N., WHEELER, D., SRIDHARAN, S., CHAKRABARTY, S., DEMMERS, J., KANAAR, R., GROTH, A. & TANEJA, N. 2023. Dynamic de novo heterochromatin assembly and disassembly at replication forks ensures fork stability. *Nat Cell Biol,* 25**,** 1017-1032.
- GERLACH, S. U., SANDER, M., SONG, S. & HERRANZ, H. 2019. The miRNA bantam regulates growth and tumorigenesis by repressing the cell cycle regulator tribbles. *Life Sci Alliance,* 2.
- HANDKE, B., SZABAD, J., LIDSKY, P. V., HAFEN, E. & LEHNER, C. F. 2014. Towards long term cultivation of Drosophila wing imaginal discs in vitro. *PLoS One,* 9**,** e107333.
- HE, S., KHAN, D. H., WINTER, S., SEISER, C. & DAVIE, J. R. 2013. Dynamic distribution of HDAC1 and HDAC2 during mitosis: association with F-actin. *J Cell Physiol,* 228**,** 1525-35.
- ITO, T. & IGAKI, T. 2021. Yorkie drives Ras-induced tumor progression by microRNA-mediated inhibition of cellular senescence. *Sci Signal,* 14.

- JAMALADDIN, S., KELLY, R. D., O'REGAN, L., DOVEY, O. M., HODSON, G. E., MILLARD, C. J., PORTOLANO, N., FRY, A. M., SCHWABE, J. W. & COWLEY, S. M. 2014. Histone deacetylase (HDAC) 1 and 2 are essential for accurate cell division and the pluripotency of embryonic stem cells. *Proc Natl Acad Sci U S A,* 111**,** 9840-5.
- KEHRLI, K., PHELPS, M., LAZARCHUK, P., CHEN, E., MONNAT, R., JR. & SIDOROVA, J. M. 2016. Class I Histone Deacetylase HDAC1 and WRN RECQ Helicase Contribute Additively to Protect Replication Forks upon Hydroxyurea-induced Arrest. *J Biol Chem,* 291**,** 24487-24503.
- KHAN, D. H., HE, S., YU, J., WINTER, S., CAO, W., SEISER, C. & DAVIE, J. R. 2013. Protein kinase CK2 regulates the dimerization of histone deacetylase 1 (HDAC1) and HDAC2 during mitosis. *J Biol Chem,* 288**,** 16518-16528.
- KLEBES, A., SUSTAR, A., KECHRIS, K., LI, H., SCHUBIGER, G. & KORNBERG, T. B. 2005. Regulation of cellular plasticity in Drosophila imaginal disc cells by the Polycomb group, trithorax group and lama genes. *Development,* 132**,** 3753-65.
- LEE, N., MAURANGE, C., RINGROSE, L. & PARO, R. 2005. Suppression of Polycomb group proteins by JNK signalling induces transdetermination in Drosophila imaginal discs. *Nature,* 438**,** 234-7.
- LI, Y., KAO, G. D., GARCIA, B. A., SHABANOWITZ, J., HUNT, D. F., QIN, J., PHELAN, C. & LAZAR, M. A. 2006. A novel histone deacetylase pathway regulates mitosis by modulating Aurora B kinase activity. *Genes Dev,* 20**,** 2566-79.
- LOPONTE, S., SEGRE, C. V., SENESE, S., MICCOLO, C., SANTAGUIDA, S., DEFLORIAN, G., CITRO, S., MATTOSCIO, D., PISATI, F., MOSER, M. A., VISINTIN, R., SEISER, C. & CHIOCCA, S. 2016. Dynamic phosphorylation of Histone Deacetylase 1 by Aurora kinases during mitosis regulates zebrafish embryos development. *Sci Rep,* 6**,** 30213.
- MARKS, P. A. 2010. Histone deacetylase inhibitors: a chemical genetics approach to understanding cellular functions. *Biochim Biophys Acta,* 1799**,** 717-25.
- MATA, J., CURADO, S., EPHRUSSI, A. & RORTH, P. 2000. Tribbles coordinates mitosis and morphogenesis in Drosophila by regulating string/CDC25 proteolysis. *Cell,* 101**,** 511-22.
- MCCLURE, K. D., SUSTAR, A. & SCHUBIGER, G. 2008. Three genes control the timing, the site and the size of blastema formation in Drosophila. *Dev Biol,* 319**,** 68-77.
- MUNOZ, S., BARROSO, S., BADRA-FAJARDO, N., MARQUETA-GRACIA, J. J., GARCIA-RUBIO, M. L., UBIETO-CAPELLA, P., MENDEZ, J. & AGUILERA, A. 2024. SIN3A histone deacetylase action counteracts MUS81 to promote stalled fork stability. *Cell Rep,* 43**,** 113778.
- NEUFELD, T. P., DE LA CRUZ, A. F., JOHNSTON, L. A. & EDGAR, B. A. 1998. Coordination of growth and cell division in the Drosophila wing. *Cell,* 93**,** 1183-93.
- PARVEEN, R., HARIHAR, D. & CHATTERJI, B. P. 2023. Recent histone deacetylase inhibitors in cancer therapy. *Cancer,* 129**,** 3372-3380.
- PILE, L. A., SCHLAG, E. M. & WASSARMAN, D. A. 2002. The SIN3/RPD3 deacetylase complex is essential for G(2) phase cell cycle progression and regulation of SMRTER corepressor levels. *Mol Cell Biol,* 22**,** 4965-76.
- REPISO, A., BERGANTINOS, C. & SERRAS, F. 2013. Cell fate respecification and cell division orientation drive intercalary regeneration in Drosophila wing discs. *Development,* 140**,** 3541-51.
- RUIZ-LOSADA, M., GONZALEZ, R., PEROPADRE, A., GIL-GALVEZ, A., TENA, J. J., BAONZA, A. & ESTELLA, C. 2022. Coordination between cell proliferation and apoptosis after DNA damage in Drosophila. *Cell Death Differ,* 29**,** 832-845.
- SCHUBIGER, M., SUSTAR, A. & SCHUBIGER, G. 2010. Regeneration and transdetermination: the role of wingless and its regulation. *Dev Biol,* 347**,** 315-24.
- SEGRE, C. V., SENESE, S., LOPONTE, S., SANTAGUIDA, S., SOFFIENTINI, P., GRIGOREAN, G., CINQUANTA, M., OSSOLENGO, G., SEISER, C. & CHIOCCA, S. 2016. A monoclonal antibody specific for prophase phosphorylation of histone deacetylase 1: a readout for early mitotic cells. *MAbs,* 8**,** 37-42.
- SUSTAR, A. & SCHUBIGER, G. 2005. A transient cell cycle shift in Drosophila imaginal disc cells precedes multipotency. *Cell,* 120**,** 383-93.

- TROUTH, A., RAVICHANDRAN, K., GAFKEN, P. R., MARTIRE, S., BOYLE, G. E., VERONEZI, G. M. B., LA, V., NAMCIU, S. J., BANASZYNSKI, L. A., SARTHY, J. F. & RAMACHANDRAN, S. 2025. The length of the G1 phase is an essential determinant of H3K27me3 landscapes across diverse cell types. *PLoS Biol,* 23**,** e3003119.
- WEIGMANN, K., COHEN, S. M. & LEHNER, C. F. 1997. Cell cycle progression, growth and patterning in imaginal discs despite inhibition of cell division after inactivation of Drosophila Cdc2 kinase. *Development,* 124**,** 3555-63.
- WORLEY, M. I., ALEXANDER, L. A. & HARIHARAN, I. K. 2018. CtBP impedes JNK- and Upd/STAT-driven cell fate misspecifications in regenerating Drosophila imaginal discs. *Elife,* 7.
- ZANG, Y., YOSHIMOTO, M. & IGAKI, T. 2025. Programmed cell senescence is required for sensory organ development in Drosophila. *iScience,* 28**,** 112048.
- ZHENG, Y., TIPTON, J. D., THOMAS, P. M., KELLEHER, N. L. & SWEET, S. M. 2014. Site-specific human histone H3 methylation stability: fast K4me3 turnover. *Proteomics,* 14**,** 2190-9.
- ZHOU, L., CHEN, Z., ZOU, Y., ZHANG, X., WANG, Z., ZHU, H., LIN, J., HUANG, Z., ZHENG, L., CHEN, J., XIE, M., ZHANG, M., ZHANG, R., ZHU, M., WANG, Z., ZHOU, H., GAO, S., YIN, Y., WU, Y. & KANG, T. 2025. ASB7 is a negative regulator of H3K9me3 homeostasis. *Science,* 389**,** 309-316.
- ZIELKE, N., KORZELIUS, J., VAN STRAATEN, M., BENDER, K., SCHUHKNECHT, G. F. P., DUTTA, D., XIANG, J. & EDGAR, B. A. 2014. Fly-FUCCI: A versatile tool for studying cell proliferation in complex tissues. *Cell Rep,* 7**,** 588-598.
