# Supplementary material for: Cell cycle dynamics regulate H3K27 and H3K9 histone modifications in Drosophila
Source: PLoS Biol. 2026 Mar 26;24(3):e3003371. doi: 10.1371/journal.pbio.3003371 (PMC13046271; doi:10.1371/journal.pbio.3003371)
Supplement: Attachment — Submitted filename: Responses to reviewers.docx [file pbio.3003371.s018.docx]

Responses to reviewers

Reviewer #1: The authors have addressed all my concerns. I am pleased to recommend publication of the manuscript.


**Reviewer #2:** The authors have addressed all of my comments, and I think it is fine not to include the analyses that I asked for in order to keep the story focused. However, the quantification in Figure 5O'-Q' is still confusing to me, because it looks as if there is less H3K27ac in early S-phase upon CBP overexpression. If I understand the response to Reviewer 1 correctly, H3K27ac intensities are not absolute values and were not normalized to G1, but to the mean H3K27ac signal across all FUCCI-defined phases (i.e., the global average). This should be made much more clear in the text and Figure legend.

**Response:**

We now added a statement to all relevant figure legends (Fig 5 and S5.1 and S5.2) that says : *Normalization was performed to the average fluorescence intensity across all FUCCI-defined phases (to avoid privileging any single phase as the normalization reference).* In addition we describe this normalization in the materials and methods: *To account for inter-sample variability, intensity values within each cell cycle phase were normalized by dividing them by the total nuclear marker signal summed across all phases (G1, early S, late S, and G2) within the same disc. This normalization provided the relative contribution of each cell cycle phase to the total signal, allowing for direct comparisons across samples and avoid privileging any single phase as the normalization reference.*

We did not find a good position in the main text that would not disrupt the flow. We thus hope that the changes to figure legends and materials and methods sufficiently address this concern.

Reviewer #4: The authors have addressed my concerns. Their resubmitted manuscript is greatly improved.
